# Assessing Arterial Patterns in the Motor Cortex With 7 Tesla Magnetic Resonance Imaging and Vessel Distance Mapping

**DOI:** 10.1002/hbm.70311

**Published:** 2025-08-05

**Authors:** Grazia Mietzner, Lilli Lümkemann, Frank Schreiber, Jascha Brüggemann, Abrar Benramadan, Marwa Al‐Dubai, Alessandro Sciarra, Christoph Knoll, Esther Kuehn, Oliver Speck, Stefanie Schreiber, Hendrik Mattern

**Affiliations:** ^1^ Department of Neurology Otto Von Guericke University Magdeburg Magdeburg Germany; ^2^ German Center for Neurodegenerative Diseases Magdeburg Germany; ^3^ Department of Biomedical Magnetic Resonance Otto Von Guericke University Magdeburg Germany; ^4^ Medicine and Digitalization—MedDigit, Medical Faculty, Univ. Dept. of Neurology Otto Von Guericke University Magdeburg Germany; ^5^ Institute of Cognitive Neurology and Dementia Research (IKND), Otto Von Guericke University Magdeburg Germany; ^6^ Hertie Institute for Clinical Brain Research (HIH) Tübingen Germany; ^7^ Leibniz Institute for Neurobiology Magdeburg Germany; ^8^ Center for Behavioral Brain Sciences Magdeburg Germany

**Keywords:** high‐resolution imaging, inter‐individual vascular variability, motor cortex vascularization, ultra‐high field, vessel atlas, vessel dominance, vessel supply patterns

## Abstract

Leveraging high‐resolution 7 T magnetic resonance imaging (MRI) and vessel distance mapping (VDM), the arterial supply patterns and dominances of the motor cortex, which could previously only be studied postmortem, were assessed in vivo and fully noninvasively. Beyond vessel patterns and dominances, the potential relation between the vascularization and the motor cortex thickness was studied. Twenty‐one healthy participants underwent 7 T MRI scans to map arterial supply and motor cortex at 0.45 mm isotropic resolution. The motor cortex vasculature was segmented manually with vessel‐specific labels. VDM was utilized to estimate the vessel‐specific supply regions and, subsequently, assess vessel patterns and dominances. Statistical tests were applied to test if the vasculature impacts mean motor cortical thickness estimates. Vessel patterns, that is the presence of supplying vessels, were classified as three‐, four‐, and five‐vessel patterns with a prevalence of 26.3%, 50.0%, and 23.7%, respectively. Vessel dominance, that is the ratio of supply volumes, of the ACA branches showed dominance of the pericallosal artery, callosomarginal artery, and equal contribution, in 34.2%, 34.2%, and 31.6% of the cases, respectively. For the MCA groups, the prevalence of precentral group dominance, central group dominances, and equal contribution was 13.2%, 34.2%, and 52.6%, respectively. Although the central and precentral groups were found in all hemispheres, the postcentral group was found in 28.9% of hemispheres with highly variable supply contribution. Statistical tests returned no significance for the effect of vessel patterns and dominances on the mean motor cortex thickness. With 7 T MRI and VDM, the motor cortex vascularization can be assessed fully noninvasively and longitudinally while providing overall good concordance with previous post mortem studies. Our comprehensive analysis of arterial motor cortex vascularization showed considerable variability between hemispheres, rendering the usage of pattern‐specific atlases and analysis more suitable than single normative representations. The successful translation from post mortem to in vivo enables the study of vascular reserve in disorders affecting the motor cortex, such as ALS, and can be translated to other brain regions and neurodegenerative diseases in the future.

## Graphical abstract

To elucidate structural patterns in human motor cortex vascularization, vessel distance mapping (VDM) is utilized to identify vessel territories from 7 T MRI. Subsequent transformation into MNI space facilitates the creation of vessel pattern‐specific atlases, enabling noninvasive mapping of each artery's supply based on the underlying vascularization.

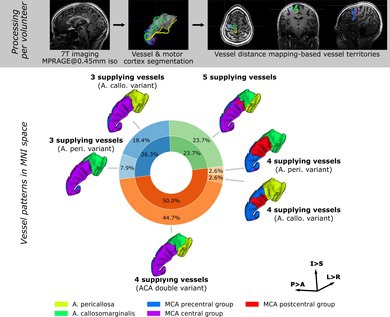

**Summary**

- This study presents the first‐ever in vivo assessment of motor cortex vascularization using 7 T MRI and vessel distance mapping (VDM), providing a comprehensive understanding of vessel patterns and dominances that were previously only accessible through postmortem expert ratings.
- Our quantitative approach, while comparable to postmortem expert ratings, surpasses previous methods by enabling the creation of 3D vessel pattern‐specific parcellations of the motor cortex beyond a single normative atlas.
- Although no significant correlation between vessel patterns, dominances, and motor cortex thickness in healthy young adults was found, the methods presented enable future studies to investigate the vascular reserve in neurodegenerative diseases like ALS and the impact of aging on the motor cortex.

## 1 Introduction

The primary motor cortex, located in the posterior frontal lobe, is essential for planning and executing voluntary movements. It is supplied by two major cerebral arteries: the anterior cerebral artery (ACA), predominantly supplying the medial part (i.e., the paracentral gyrus), and the middle cerebral artery (MCA), predominantly supplying the lateral part (i.e., the precentral gyrus). However, as shown post mortem, there are considerable inter‐individual differences in the arterial supply of the primary motor cortex (Ugur et al. 2005). In general, the vasculature shows considerable inter‐individual differences throughout the brain, and assessment of these vascular configurations is important beyond neuroanatomical description. For example, the configuration of the Circle of Willis, which is the main cranial pathway of collateral arterial circulation, is related to the outcome after stroke (Oumer et al. 2021; Westphal et al. 2021).

In cerebral small vessel diseases (CSVD), the hippocampal vessel patterns, that is whether the hippocampus is solely supplied by the posterior cerebral artery or additionally from the anterior choroidal artery, govern resilience towards cognitive decline (Perosa et al. 2020; Vockert et al. 2021). Further, significant differences in anterior hippocampal and total gray matter volume were related to the hippocampal supply pattern in a cross‐sectional study of CSVD patients and controls (Vockert et al. 2021). Thus, the hippocampal patterns contribute to maintaining structural integrity beyond the medial temporal lobe (Vockert et al. 2021). This concept of vascular patterns and reserve could translate to the motor cortex, that is for diseases such as amyotrophic lateral sclerosis (ALS) (Schreiber et al. 2023). In addition, a recent study showed that cortical aging in the primary motor cortex is dependent on the distance to the nearest vein (Knoll et al. 2024). However, to study the inter‐individual differences in motor cortex vascularization and their relation to aging and pathology, we first have to provide a precise description of the arterial supply patterns that can be mapped in healthy participants in vivo, which have only been studied post mortem to date.

More precisely, in a post mortem study investigating 40 hemispheres of healthy adults, Ugur et al. showed that the ACA supplies the motor cortex through the pericallosal artery (A. pericallosa) and the callosomarginal artery (A. callosomarginalis), whereas the supply by the MCA can be categorized into a precentral, central, and postcentral group (Ugur et al. 2005). In line with other brain regions, such as the hippocampus (Spallazzi et al. 2018; Erdem et al. 1993) inter‐individual differences in the supplying vessels were found; that is, a single ACA branch (A. pericallosa or A. callosomarginalis) can dominate the motor cortex supply, but also both ACA vessels can contribute equally to the supply. Analogously, the motor cortex is supplied by the precentral and central MCA group, or additionally by the postcentral group (Ugur et al. 2005). Therefore, systematic inter‐individual differences in the supplying vasculature of the motor cortex exist, but have so far not been mapped out precisely using in vivo imaging.

However, for the future assessment of potential reserve mechanisms of the motor cortex, or to characterize cortical aging in reference to the individual vascular architecture, first, the vessels have to be imaged and assessed non‐invasively and in vivo. In line with past efforts by Spallazzi et al. to establish the hippocampal vessel patterns in vivo (Spallazzi et al. 2018), we here leverage the high‐resolution capabilities of 7 Tesla Magnetic Resonance Imaging (7 T MRI) to enable, for the first time, the in vivo assessment of individual differences in the motor cortex vascularization. We apply vessel distance mapping (VDM), a novel postprocessing technique used previously to study hippocampal vessel patterns (Garcia‐Garcia et al. 2023), to assess quantitatively the vasculature. Compared to expert ratings, VDM enables the data‐driven estimation of vessel supply territories to generate 3D supply atlases of the motor cortex. In addition, VDM is leveraged to assess vessel patterns and vessel dominances. Although vessel patterns, that is the number of individual supplying arteries, were used in previous in vivo MRI studies of the hippocampal vascularization (Perosa et al. 2020; Vockert et al. 2021; Spallazzi et al. 2018), vessel dominances, that is the supply contribution equal between vessels or does one vessel dominate, was used by Ugur et al. to study the motor cortex vascularization post mortem (Ugur et al. 2005). By integrating both of these potentially complementary concepts, this study aims to assess the impact of the supplying vascularization on cortical thickness in healthy volunteers. This particular focus on the relationship between vasculature and cortical structure is motivated by prior research, including Vockert et al. (2021) findings on differences in gray matter volumes associated with hippocampal vessel patterns Bernier et al. (2018) observation that cortical thickness correlates with vessel density across the cortex.

## 2 Materials and Methods

**FIGURE 1:**
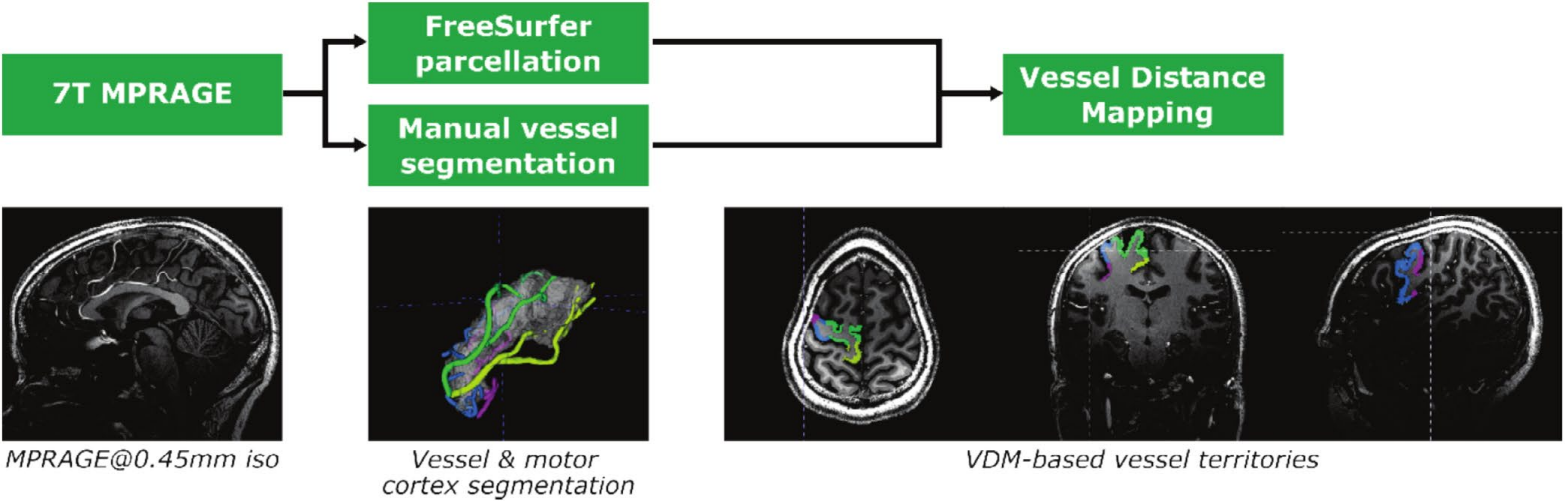
Overview of image processing: The anatomical and vascular information inherently captured by high resolution 7 T MPRAGE data is used to automatically parcellate the brain with FreeSurfer and manually label the arteries (individual label/color per artery). Subsequently, vessel distance mapping (VDM) is applied to compute for each voxel in the motor cortex which is the closest segmented arterial segment. This enables the data‐driven, distance‐based, parcellation of the motor cortex into vessel territories.

In the following, the sequences to acquire 7 T MRI data, the methods to segment the motor cortex and its vasculature, the tools to compute VDM‐based vessel territories, and the statistical assessment of the vessel patterns, dominance, and potential effect on cortical thickness are reported. A schematic overview is provided in Figure 1.

### 2.1 7 T MRI Data Acquisition

In this study, which was approved by the local ethics committee (local IRB number 28/07), *n* = 21 volunteers (14 males, 31.5 ± 6.1 years, and 7 females, 27.3 ± 3.4 years) without any known neurological disorder were included after giving written and informed consent. Data were acquired with the original model MAGNETOM 7 T MRI (Siemens Healthineers, Erlangen, Germany; software version VB17) equipped with a 1Tx/32Rx‐head coil (Nova Medical, Wilmington, MA, USA). One prospectively motion‐corrected MPRAGE at an isotropic resolution of 0.45 mm was acquired per volunteer with the following parameters: 3D encoding matrix of 496 × 496 × 416, echo time of 2.82 ms, inversion time of 1050 ms, repetition time of 2820 ms, readout bandwidth of 170 Hz/voxel, 5° flip angle, acceleration with GRAPPA factor 2, total scan time of 12:12 min.

Note that the motion‐corrected MPRAGE data used here is a subset of the study by Sciarra et al. (Sciarra et al. 2022) in which the motion correction performance of an optical tracking system was evaluated for four different MR image contrasts. Overall, image quality improved when correcting for motion compared to uncorrected data acquisition (Sciarra et al. 2022). Further, previous studies showed that vessel depiction and sharpness improved when using the optical tracking system to prospectively correct motion (Mattern et al. 2018, 2019).

Due to prolonged T1 relaxation times at ultra‐high field MRI, MPRAGE data acquired at 7 T provide good arterial depiction, outperforming Time‐of‐Flight angiography, the default for noncontrast enhanced arterial MRI, for larger‐ to medium‐sized vessels (Maderwald et al. 2008). With the additionally used prospective motion correction and the high isotropic resolution of 0.45 mm, the MPRAGE data used here provide in a single scan structural and vascular contrast to enable the segmentation of the motor cortex and arteries of interest. Given the cortex and vessel segmentation data are hence inherently aligned, rendering additional co‐registration steps obsolete.

### 2.2 Assessment of the Motor Cortex and Its Arterial Vasculature

From the MPRAGE data, the motor cortex was automatically segmented by using FreeSurfer (Fischl 2012). To that end, FreeSurfer's masks for the medially located paracentral gyrus and laterally located precentral gyrus were merged per hemisphere, resulting in one mask per hemisphere encompassing the entire motor cortex. In addition, FreeSurfer was used to estimate the cortical thickness of the precentral gyrus, paracentral gyrus, and motor cortex, respectively.

Within Mango (Multi‐image Analysis GUI provided by the University of Texas Health Science Center at San Antonio), all arteries of interest were delineated fully manually (identified for each voxel in every slice without automation support like region growing), each with its own label per hemisphere: the A. pericallosal and A. callosomarginalis of the ACA and the precentral group, central group, and postcentral group of the MCA. Only vessel segments that reached the region of interest (ROI), that is motor cortex mask, were delineated with the label of their respective branch/group. The manual vessel delineation in both hemispheres of all volunteers was performed by G.M. (4 years of arterial vessel delineation experience). For a subset of six hemispheres, L.L. (3 years of arterial vessel delineation experience) performed delineations to assess rater agreement. More details on the vessel identification and manual delineation are provided in the (see Figure S1).

In addition to the delineation and toward a holistic assessment of the motor cortex vasculature, the ACA and MCA bifurcations were assessed manually. Per parent vessel, that is ACA or MCA, the bifurcations were tracked from the vessel's origin to the motor cortex in sagittal views. This enabled the assignment of a bifurcation level to each vessel going to the motor cortex. The analysis of the bifurcation level and frequencies of subbranches going to the motor cortex was carried out for the A. pericallosal, A. callosomarginalis, precentral group, central group, and postcentral group. Further, the arterial radii were estimated, and their relation to distance to the motor cortex and potential differences in vessel radii across vessel patterns were assessed (see Supporting Information).

### 2.3 Data‐Driven Estimation of Supply Territories With VDM

**FIGURE 2:**
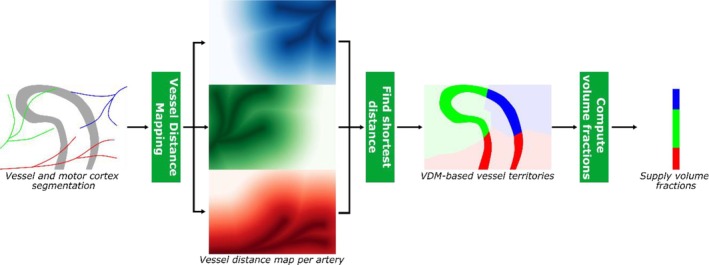
Illustration of the VDM‐based workflow to estimate vessel territories and supply volume fractions. Per segmented artery (colored lines) around the motor cortex (gray, curved structure), a vessel distance map is generated by computing voxel‐wise the Euclidian distance to the closest artery segment of interest. Subsequently, per voxel the shortest distance across all distance maps is found and the respective artery label assigned. Using the volume of the supply territory with in the region of interest, supply volume fractions are computed.

**FIGURE 3:**
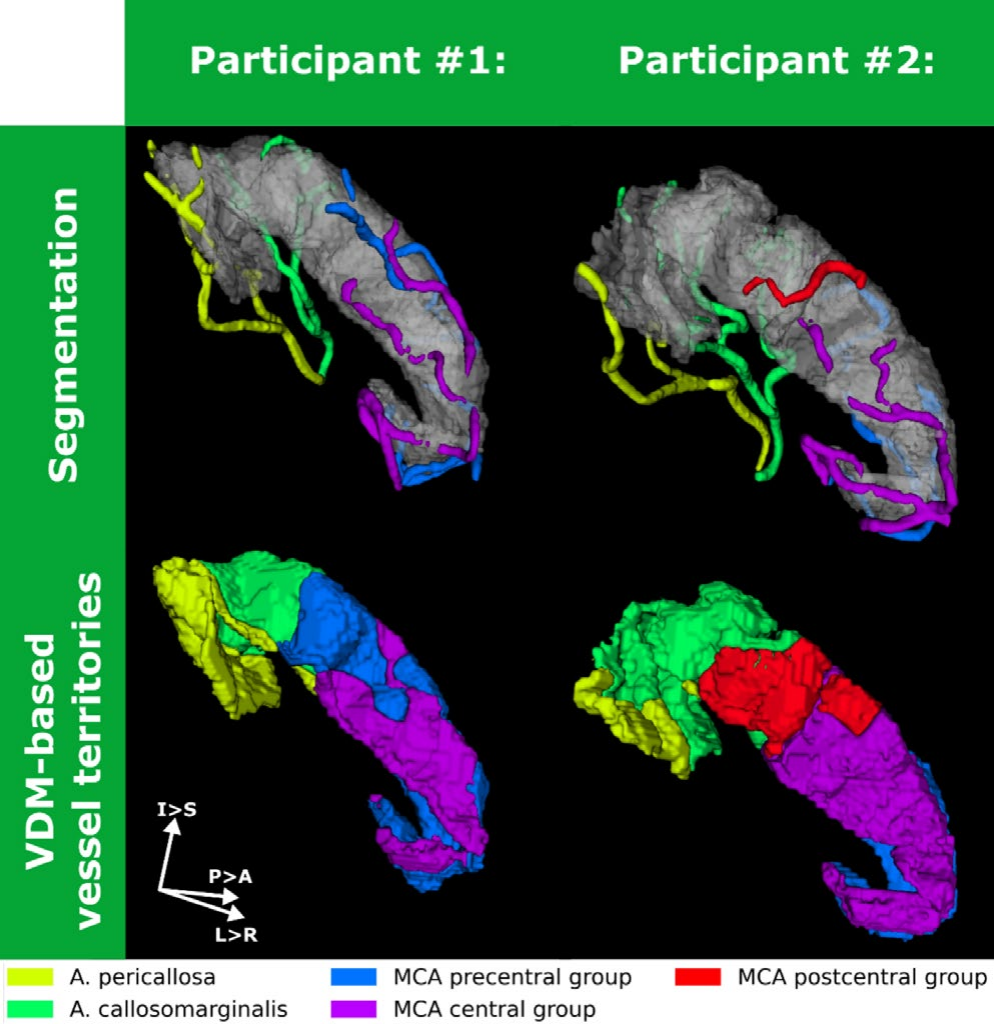
Vessel delineation and segmentation of motor cortex: Comparison of vessels and their VDM‐based vessel territories for two representative right hemispheres showing inter‐individual differences in vessel trajectories, motivating the investigation of arterial motor cortex patterns. Beyond the present MCA postcentral group, participant #2 has a considerably larger contribution by the A. callosomarginalis than A. pericallosa compared to participant #1. Further, the MCA's central and precentral group shows distinct differences.

Supply territories were approximated from the vessel delineations with a VDM‐based approach (implemented in Python, code available at https://github.com/hendrikmattern/VesselDistanceMapping.git) (Knoll et al. 2024; Garcia‐Garcia et al. 2023; Mattern et al. 2021). VDM computes the shortest distance to the segmented vasculature per voxel. Here we additionally computed distance maps for each artery individually. Hence, per hemisphere and voxel, the distance to the A. pericallosal, A. callosomarginal, precentral, central, and postcentral group is computed (if vessel not present/delineated, VDM for the respective artery is skipped and respective supply volume set to zero). Therefore, for each voxel up to five distance estimates were available and for the supply territories, the label of the closest artery was assigned. With this “winner takes all” approach (only shortest artery considered, relative differences in closeness of arteries not considered), the motor cortex (only gray matter as estimated by FreeSurfer's parcellation) was parcellated into supply territories based on the voxel‐wise vessel distances (see Figure 2 for an illustration of the workflow and Figure 3 for representative examples). Besides the 3D‐resolved supply territories, the relative size of the supply territory was estimated per hemisphere. These supply volume fractions of the motor cortex's gray matter were the basis for subsequent vessel pattern and dominance assessment.

Note that this VDM‐based approach resembles the data‐driven estimation of vascular territories of the anterior cerebral artery, middle cerebral artery, and posterior cerebral artery presented by Mut et al. although no probabilistic atlas or multiple‐seed region growing technique was used here (Mut et al. 2014). Although both data‐driven approaches are not based on vessel‐specific perfusion measurements, they provide parcellation of the brain into supply territories, similar in style to representations commonly used in textbooks.

### 2.4 Vessel Patterns and Dominance Assessment With VDM

Motivated by previous studies of hippocampal vessel patterns (Perosa et al. 2020; Vockert et al. 2021; Garcia‐Garcia et al. 2023), we define vessel patterns by the number of independent arteries supplying the ROI with a maximum of five vessels per hemisphere (up to two ACA branches and three MCA groups involved). Vessel patterns were estimated by counting the number of nonzero supply volume fractions per hemisphere, and the (relative) frequencies of each pattern are reported. In addition to the number of independent arteries, vessel patterns were stratified further by the individual ACA branches and MCA groups contributing to the pattern. As an example, a pattern with four supplying vessels could be realized by two ACA branches and two MCA groups or one ACA branch and three MCA groups. Hemisphere‐wise vessel pattern results are reported for the entire motor cortex (see results), precentral gyrus, and paracentral gyrus (see Supporting Information).

In addition to pattern frequencies, the average supply volume fractions per vessel pattern are reported, and pattern‐specific supply territory atlases were generated. To generate atlases in MNI space, the following steps were performed: First, all hemispheres have been co‐registered nonlinearly to the left hemisphere of the ICBM 2009b nonlinear, asymmetric MNI template (Fonov et al. 2011, 2009). To that end, the MPRAGE images were bias field‐corrected with N4 (Tustison et al. 2010) prior to the co‐registration with ANTs (antsRegistrationSyN.sh with rigid, affine, and deformable SyN registration) (Avants et al. 2008). For all right hemispheres, a left–right flip of the brain was performed prior to the co‐registration so that the right hemispheres were co‐registered to the left hemisphere of the asymmetric template. Subsequently, all vessel labels and motor cortex masks were transformed from their respective native space into the common MNI space by applying the transform estimated in the co‐registration step of the MPRAGE images (nearest neighbor interpolation). Afterwards, all anatomical masks were merged using antsJointFusion (Wang et al. 2013; Wang and Yushkevich 2013) to generate one representative ROI mask in MNI space. Lastly, 3D supply territory atlases of the motor cortex in MNI space were generated. As mentioned above, the motor cortex of all hemispheres was parcellated into 3D supply territories by assigning each voxel the label of the vessel with the shortest distance. These vessel supply territories were merged in MNI space by selecting the most frequent vessel label per voxel. This process was performed for each vessel pattern individually to generate pattern‐specific vessel supply territory atlases. These 3D parcellations of the motor cortex enable a qualitative comparison in 3D of the vessels' supply territories per vessel pattern. In addition to supply territory atlases, the vessel‐specific average distances in MNI space were computed by linearly interpolating the vessel distances into MNI space and computing a voxel‐wise average (see Figure S2).

To quantify the relative contribution of each vessel, we additionally computed vessel dominances (VDom) in the native MPRAGE space of each volunteer. Therefore, two supply volume fractions were put into relation with each other by computing the ratio of the two supply volume fractions. In more detail, to determine if a certain vessel is dominating, the volume supplied by the vessel of interest should be considerably larger than the volume supplied by its counterpart vessels. The ratio of the supply volume of interest to the sum of the supply volume of interest plus its counterpart volume is 1.0 if the vessel of interest is the only supplying vessel, 0.5 if there is equal contribution by both, and 0.0 if the vessel of interest is not contributing. The following ratios have been computed:

- ACA‐MCA‐ratio, that is the ratio of the ACA volume fraction to the entire motor cortex volume, to quantify the contribution of the ACA branches.
- Pericallosa‐callosomarginalis‐ratio, that is the ratio of the pericallosa volume fraction to the entire ACA supply volume, to quantify A. pericallosa (ratio close to 1.0) versus A. callosomarginalis dominance (ratio close to 0.0).
- Central‐precentral‐ratio, that is the ratio of central group volume fraction to combined central and precentral group volume fraction, to quantify central group (ratio close to 1.0) or precentral group dominance (ratio close to 0.0). The postcentral group was not considered here as it is only present in less than 30% of the hemispheres and accounted for only a small volume fraction.
- Postcentral‐MCA‐ratio, that is ratio of the postcentral group volume fraction to the entire MCA supply volume, to quantify the postcentral group's contribution to the entire MCA branches supply.

Besides the complementary nature of vessel dominance, that is supply volume ratios, and vessel patterns, that is number of independent supplying arteries, the VDom estimates enabled a comparison to the post mortem study of vessel dominance by Ugur et al. For this comparison, the continuous ratios of our in vivo method had to be converted into qualitative ratings as reported in the post mortem benchmark study (Ugur et al. 2005), that is single vessel dominant versus equal contribution. For this conversion, a lower and upper threshold have been employed and the frequency of vessel dominance is reported. Ratios in between the lower and upper threshold are considered equal contribution while ratios below the lower or above the upper threshold indicate single vessel dominance. The upper and lower thresholds were varied systematically and jointly (100 lower thresholds equally spaced in the interval of 0.05–0.45 and the respective upper threshold computed as 1 minus lower threshold returning a range of 0.95–0.55) to validate the robustness of VDom ratings and test whether the equidistant threshold choice (0.33, 0.66) provided similar results to thresholds yielding highest agreement with the post mortem data by Ugur et al. (Ugur et al. 2005). Note that 0.33 and 0.66 for the lower and upper threshold represent equally spaced thresholds and any ratio in between these thresholds would be considered an equal contribution of both vessels.

### 2.5 Statistical Assessment

Descriptive results are reported as mean ± standard deviation unless stated differently, and processing was performed with Python 3.9, scipy 1.12.0, statsmodel 0.14.1, and pingouin 0.5.4.

Prior to testing the effect of vessel patterns and vessel dominance on average thickness of the motor cortex, differences in cortical thickness due to covariates, that is left or right hemisphere, sex, and age, were tested. Further, group differences in artery radii for different vessel patterns were assessed (results shown in Supporting Information).

Based on normality, homoscedasticity, and the number of groups, the appropriate statistical tests for categorical independent variables were selected for the covariates, vessel radius estimates, and inter‐hemispheric differences, that is paired and unpaired *t*‐Test, one‐way ANOVA, one‐way Welch ANOVA, or nonparametric Kruskal‐Wallis *H*‐Test. For continuous independent variables, linear regression with Pearson correlation was performed.

Motivated by past studies showing vessel patterns and vessel density relate to cortical thickness (Vockert et al. 2021; Sciarra et al. 2022), we performed model comparisons using ordinary least squares (OLS) regression to assess the effect of vasculature on the thickness of the motor cortex. Given the significant correlation of age and sex, but not laterality, with cortical thickness (see Supporting Information), age and sex were included as covariates. The following test strategy was performed for vessel patterns, ACA branch dominance, and MCA group variance, respectively: (i) A full model including age, sex, and the vasculature metric (modeled as a categorical variable) was fitted to predict the cortical thickness estimates. This yields the contribution, that is slope, and *p* value per independent variable, that is vasculature metric and covariates. (ii) To infer if the addition of the vascular metric significantly improved the explanatory power, a reduced model containing only the covariates age and sex was constructed, and the full and reduced model were compared via ANOVA. Note that all OLS regressions used robust standard errors (HC3) to account for potential heteroscedasticity. Additionally, adjusted *R* ^2^ values for all models were computed.

For visual group comparison of vasculature and cortical thickness, the thickness estimates were adjusted for age and sex prior to plotting by adding residuals from the reduced model to its intercept, preserving baseline levels while removing linear covariate effects. Adjusted thickness values were grouped by vessel pattern or dominance category and plotted.

## 3 Results

After delineating the vessels for all 21 volunteers, two volunteers were identified in which one hemisphere had a bilateral supply from the ACA, that is the right motor cortex was supplied by the left A. pericallosa in addition to the vessels from the right hemisphere. Although Ugur et al. reported one case with cross‐hemisphere vascularization as well (Ugur et al. 2005), the two volunteers were excluded from the further hemisphere‐wise analysis. After exclusion, the average age of the remaining 19 volunteers (7 females) was 31.18 ± 6.48 years.

In the following, the results for the remaining 38 hemispheres are presented. First, results on vessel segmentation and branching are reported, followed by the analysis of vessel patterns and dominances. Finally, results on the potential effect of the vascularization on cortical thickness in healthy volunteers are reported.

### 3.1 Motor Cortex Vascularization and Segmentation

The motor cortex vascularization was successfully identified in 7 T MRI MPRAGE images. In line with previous post mortem studies, we observed considerable variability in how arteries propagate to the motor cortex. Although the precentral and central groups of the MCA were found around the motor cortex in all hemispheres, the postcentral group was only present in 11 out of 38 hemispheres. For the ACA, the A. pericallosa and A. callosomarginalis were found in 34 and 30 out of the 38 hemispheres, respectively. Further details on the bifurcation and subbranches of the motor cortex vascularization are provided in the (see Figure S3).

Besides the presence of arteries itself, their respective contribution, that is VDM‐based supply territory, is variable as well. To illustrate this and to support the following vessel pattern and dominance assessment, two representative hemispheres with different vessel configurations are shown in Figure 3. From the manual, vessel‐specific delineations, VDM is used to estimate the supply territories of each artery. Beyond the postcentral group, which is only present in one of the two hemispheres, variations in the territories can be perceived qualitatively and quantitatively by estimating the volume fraction of the motor cortex assigned to each vessel, that is supply volume fractions. Although the supply territory by ACA branches in total is similar in both hemispheres (volume fractions of 0.412 vs. 0.445 for left and right column, respectively), there is a 3‐fold difference between the A. pericallosa contribution (volume fractions of 0.182 vs. 0.055 for left and right column, respectively). Hence, while in both hemispheres the two ACA branches contribute to the supply, that is vessel pattern, the contribution per vessel varies considerably, that is vessel dominance. These observations for the ACA branches translate to the MCA groups. Although the overall MCA supply is similar across both hemispheres (volume fractions of 0.588 vs. 0.554 for left and right column, respectively), the contribution of the MCA group varies considerably. For the precentral and central group, the volume fraction is approximately twice as much in one compared to the other hemisphere (volume fractions of 0.431 vs. 0.208 and 0.157 vs. 0.275 for precentral and central group, respectively). Additionally, the postcentral group is only present in one of the two hemispheres. The here‐found inter‐hemispherical variability in vessel territories for two representative hemispheres will be investigated for all 38 hemispheres with respect to vessel pattern and vessel dominance. The basis of pattern and dominance assessment are the supply volume fractions per vessel, which are shown in the (see Figure S4), that is per hemisphere the relative volume fraction of the ROI assigned to each vessel of interest is shown.

To test the robustness of our volume fractions, for a subsample of six hemispheres, the vessel delineation was repeated by a second rater. Subsequently computed supply volume fractions showed a high and significant Pearson correlation between both raters (*r* = 0.966, *p* < 1e‐17, for details see, Figure S5).

### 3.2 Vessel Patterns

Comparing the supply volume fractions for all hemispheres (see Figures S4 and S5) reveals the following general trends: The ACA branches, that is A. pericallosa and A. callosomarginalis, and MCA groups, that is precentral, central, and postcentral group, account for approximately 40% and 60% of the motor cortex supply, respectively, while the contribution of the individual branches and groups is highly variable. For the precentral gyrus, the values are 24% and 76% for the ACA branches and MCA groups, respectively, whereas the paracentral gyrus is exclusively supplied by ACA branches.

**FIGURE 4:**
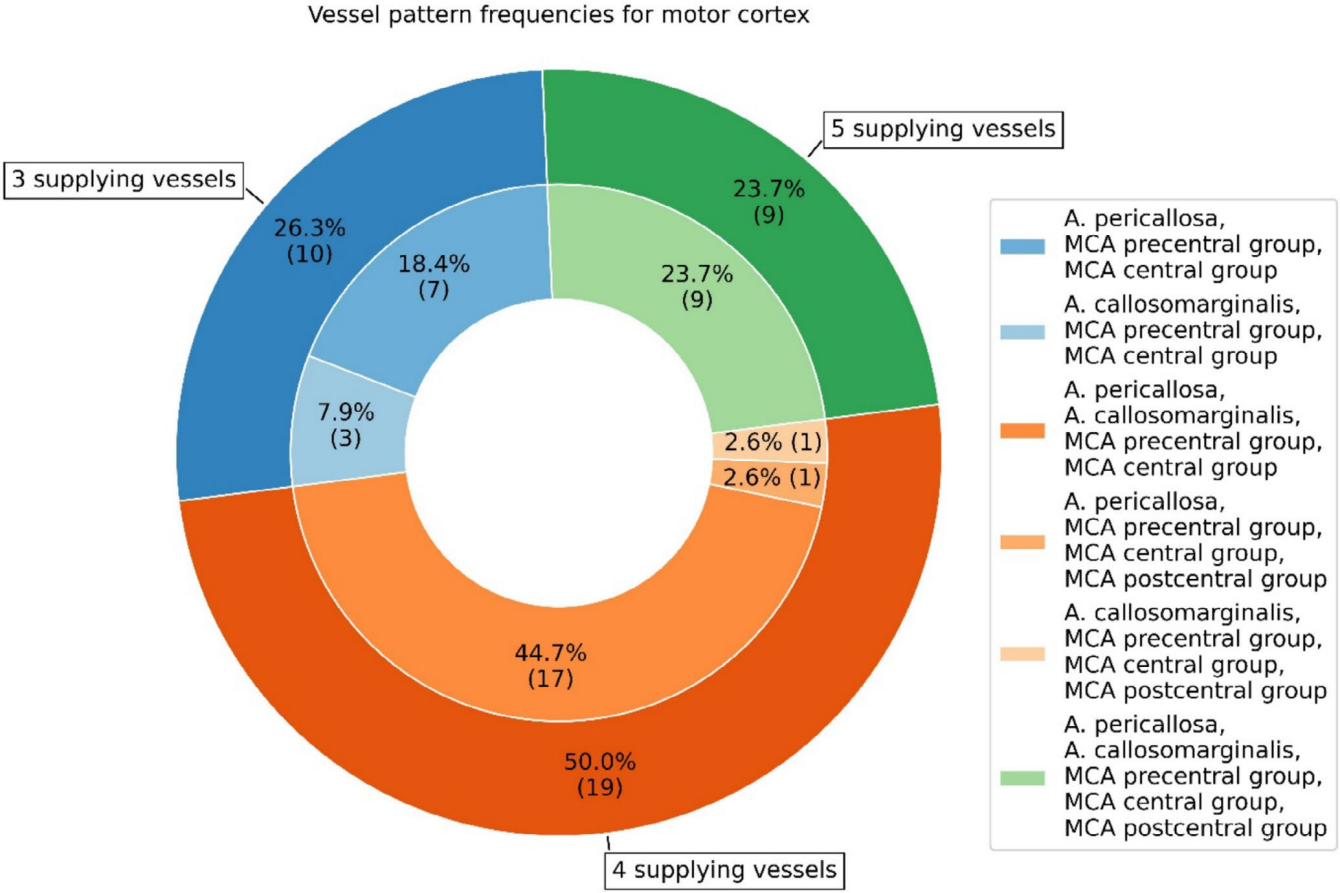
Prevalence of vessel supply patterns in the motor cortex as estimated with VDM (fractions provided in percent, absolute count in parenthesis).

The prevalence of vessel patterns, that is the number of vessels with a nonzero supply volume fraction, was computed for the motor cortex (see Figures 4 and Figure S6 for patterns of the precentral and paracentral gyrus):

Our data‐driven assessment returns vessel patterns with three, four, or five distinct vessels involved in the supply of the motor cortex. Prevalence for four supplying vessels was approximately twice as high as for three and five vessels (26.3%, 50.0%, and 23.7% for three, four, and five vessels, respectively).

Out of the 19 cases with four supplying vessels, 17 had a vessel configuration involving both ACA branches and the MCA precentral and central group. The remaining two hemispheres had all MCA groups, that is precentral, central, and postcentral group, and one of the two ACA branches supplying the motor cortex. For the 10 hemispheres supplied by three vessels, the supplying vessels were always the precentral and central group of the MCA along with one of the two ACA branches (7 and 3 cases for A. pericallosa and A. callosomarginalis, respectively). The remaining nine hemispheres were supplied by all five vessels. The postcentral group only was found in supply patterns with four or five vessels (2 and 9 out of 11 hemispheres with postcentral group for four‐ and five‐vessel pattern, respectively). Average supply volume fractions per pattern are provided in the (see Table S1).

**FIGURE 5:**
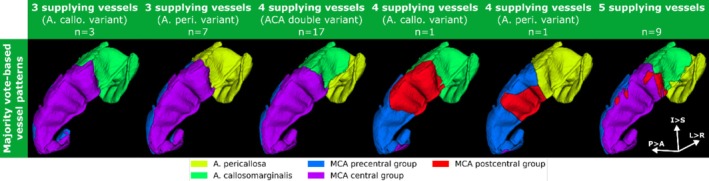
3D renderings of the six vessel patterns in MNI space. After co‐registering all hemispheres to the same space, for each voxel the most frequent artery label, that is majority vote, was assigned. Although the border between the ACA and MCA vessels is relatively stable across patterns, the contribution of individual vessels is highly variable across patterns.

Beyond pattern frequencies, the hemisphere‐wise supply territories were transformed into MNI space to generate vessel pattern‐specific parcellations of the motor cortex, that is supply territory atlases (see Figure 5 for 3D renderings and Figure S7 for representative axial views). The split between ACA and MCA territories is considerably stable across all six different patterns. In line with the results reported above, the paracentral gyrus is exclusively supplied by the ACA branches. For patterns having competing vessels of the same parent vessel, that is the central versus precentral group or A. callosomarginalis versus A. pericallosa, their respective borders are similar across patterns (e.g., precentral vs. central group in all 3 supplying vessels variants and 5 vessel pattern). Although no true perfusion measurement, this could indicate that watershed areas are similar across patterns. Although the precentral and central groups are present in all patterns, the two ACA branches and postcentral group do not always contribute. Since the overall ACA territory is rather stable across patterns, in a single ACA branch pattern, the entire region of the motor cortex attributed to the ACA is supplied by the single branch. In the two hemispheres with four vessels and the postcentral present, the postcentral group supplied a considerable part of the precentral cortex while the central group is almost absent (small portion at the interior slices of the motor cortex). For the five‐vessel pattern, the postcentral group also becomes negligible in size.

Overall, the vessel patterns of the motor cortex reconstructed via majority votes in MNI space can be understood as the superposition of the individual precentral‐central‐group pattern and callosomarginalis‐pericallosa pattern and their associated borders. Only two out of 39 cases with four vessel and postcentral contribution represent variants distinctively different from the others w.r.t. MCA supply territories.

Bilateral vessel atlases in the MNI152 NLIN 2009b space are publicly available (Mattern et al. 2025).

### 3.3 Vessel Dominance

The trend of considerable inter‐hemispheric variability is also observed for vessel dominances. Although computing the average volume ratios yields approximately equal contributions for the ACA branches and MCA groups, there is considerable variance across the 38 hemispheres (volume ratio of A. pericallosa to entire ACA supply of 0.518 ± 0.344, ratio of central group to MCA supply excluding postcentral group of 0.550 ± 0.225). Hence, considering the average VDom conceives the inter‐hemispheric variance, which stems from the previously estimated and highly variable supply volume fractions. Therefore, the corresponding ratio, that is VDom estimates, is highly variable as well.

For comparison with the qualitative rating obtained in the post mortem study by Ugur et al. (Ugur et al. 2005), the continuous volume ratios were converted into ratings by applying a lower and upper threshold. In the following, the equidistant thresholds, that is 0.33 and 0.66, are applied, with a volume ratio between 0.33 and 0.66 being considered as equal contribution of the two vessels of interest. A detailed assessment of the effect of thresholds on VDom ratings is provided in the (see Figure S9 and Table S3).

**FIGURE 6:**
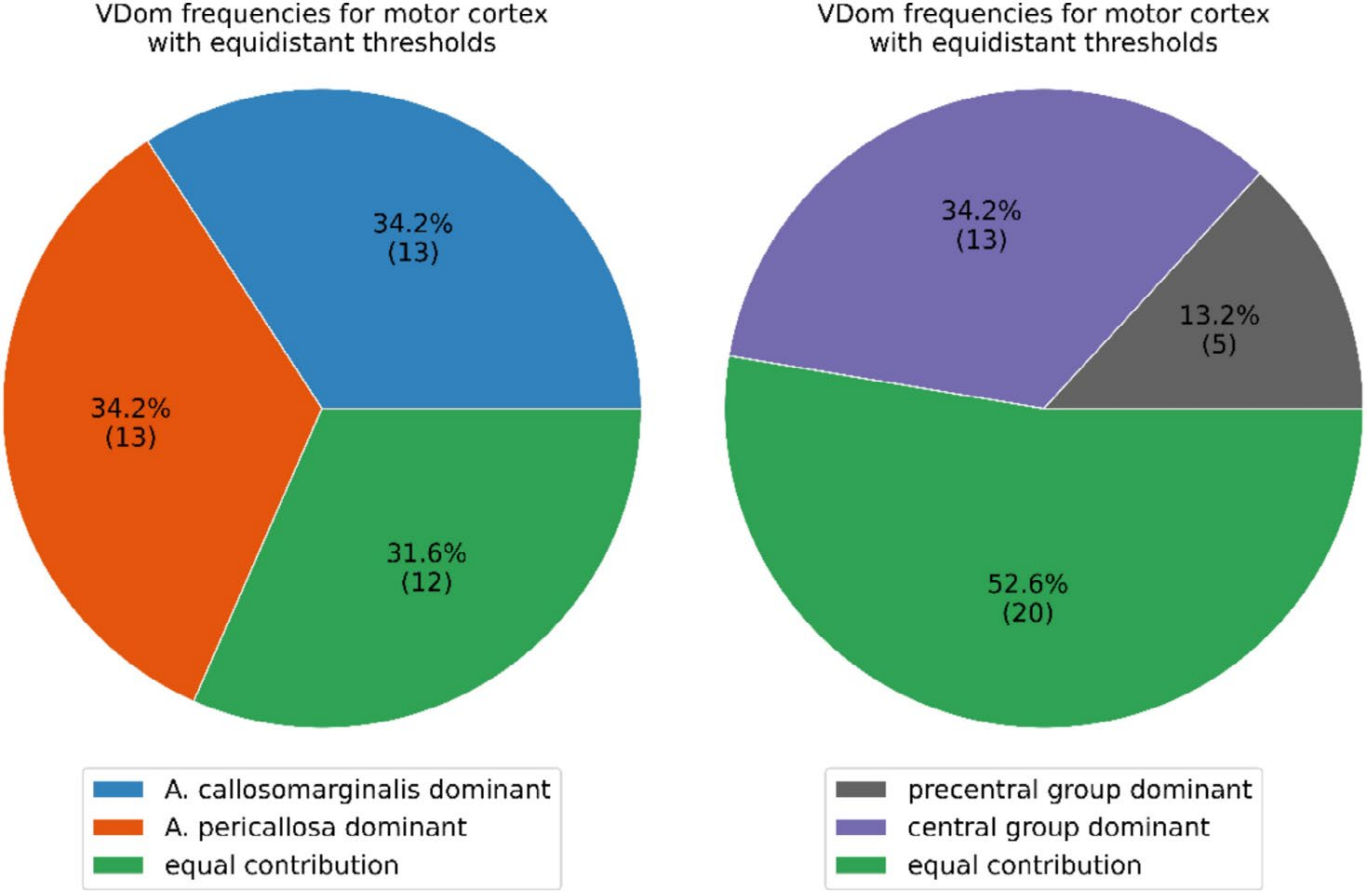
Vessel dominance (VDom) for the ACA branches, that is A. callosomarginalis and A. pericallosa, and MCA groups, that is precentral and central group (fractions provided in percent, absolute count in parenthesis). To convert the continuous volume ratios into ratings for dominance, equidistantly spaced thresholds are applied with a volume ratio between 0.33 and 0.66 being considered an equal contribution of both vessel branches/groups.

For the ACA branches, an almost even split of A. callosomarginalis dominance, A. pericallosa dominance, and equal contribution can be observed (34.2%, 34.2%, and 31.6%, respectively; see Figure 6). For the MCA group, equal contribution by the precentral and central group is observed for more than half of the hemispheres (52.6%; see Figure 6). For single vessel group dominance, the central group is more than twice as likely to be dominant than the precentral group (34.2% vs. 13.2%). Note that the postcentral group was not considered for the VDom rating as it was only present in 11 of the hemispheres, and its supply volume ratio was small and highly variable (0.035 ± 0.070%). All volume ratios are summarized in tabular form in the (see Table S2).

Comparison of the ACA VDom ratings obtained with the equidistant threshold and vessel dominance estimates provided by Ugur et al. yield the following results: A. pericallosa dominant 34.2% versus 32.5%, A. callosomarginalis dominant 34.2% versus 40.0%, equal contribution 31.6% versus 27.5% (Ugur et al. 2005). Although details on the effect of the threshold on VDom ratings and the agreement with Ugur et al. are provided in the (see Figure S9), the main findings can be summarized as follows: The equidistant threshold and the one optimized for agreement with Ugur et al. are virtually identical (0.33 and 0.66 vs. 0.34 and 0.66 for equidistant and optimized). In between 0.3–0.4 and 0.6–0.7 for the upper and lower threshold, respectively, changes in the obtained VDom frequencies were arguably small (see Figure S9).

For the MCA groups, the overall agreement was suboptimal, regardless of using equidistant or thresholds optimized for agreement with Ugur et al. (Ugur et al. 2005). Using the equidistant threshold, the comparison to Ugur et al. yields: central group dominant 34.2% versus 72.5%, precentral group dominant 13.2% versus 10.0%, equal contribution 52.6% versus 17.5% (Ugur et al. 2005) (see Supporting Information for results optimized for agreement with Ugur et al.). Although the precentral dominance estimates in vivo and post mortem agree, the central group dominance is considerably lower in our study, resulting in more equal contribution ratings. Potential sources of this systematic bias are discussed below. We did not explicitly compute postcentral group dominance as the associated supply volume fractions were overall low. Hence, without any computation, it can be seen that the postcentral group is never dominant, which is in line with Ugur et al. (Ugur et al. 2005).

### 3.4 Vasculature and Cortical Thickness

While no significant difference between left and right hemisphere in mean cortical thickness of the motor cortex was found [*t*(36) = −0.854, *p* = 0.399, *d* = 0.277], a significant difference and correlation were observed for sex [*t*(24.561) = −4.607, *p* < 0.001, *d* = 1.604] and age [*r*(37) = −0.384, *p* = 0.035], respectively (more details provided in see Figure S11).

To assess the relationship between motor cortex thickness and vasculature, we employed ordinary least squares (OLS) regression, controlling for age and sex, and performed model comparisons using ANOVA, that is tested if the addition of the vasculature in the full model improved predictive power significantly compared to the reduced model which included only the covariates.

In all OLS models, sex was the dependent variable with the strongest coefficient and always returned significance. Age was always the dependent variable with the lowest coefficient and never reached significance. For the full models, only the coefficient representing the difference between 3‐ and 4‐supplying vessels reached significance (*p* = 0.042), whereas the coefficient for the difference between 4‐ and 5‐supplying vessels might be indicative of a trend but did not reach significance (*p* = 0.096). The coefficients for VDom did not reach significance or considerable low *p* values (*p* = 0.647 and *p* = 0.858 for ACA and MCA VDom, respectively). All statistics are provided in the Supporting Information.

When comparing models including the number of supplying arteries to the reduced model, no significant improvement in model fit was found [*F*(2, 33) = 2.233, *p* = 0.123], although the adjusted *R* ^2^ increased from 0.354 to 0.397. Mean cortical thickness (±SD) for individuals with 3, 4, and 5 supplying vessels was 2.566 ± 0.047 mm, 2.512 ± 0.081 mm, and 2.531 ± 0.038 mm, respectively. Similarly, models including ACA branch dominance or MCA group dominance (equidistant thresholds) did not significantly improve fit over the reduced model [ACA VDom: *F*(1, 34) = 0.230, *p* = 0.635; MCA VDom: *F*(1, 34) = 0.040, *p* = 0.842]. In both cases, adjusted *R* ^2^ values slightly decreased compared to the reduced model (0.354 for the reduced model and 0.340 and 0.336 for full model with ACA VDom and MCA VDom, respectively). Mean cortical thickness for single vessel dominance versus equal contribution was 2.534 ± 0.066 mm versus 2.523 ± 0.074 mm and 2.529 ± 0.061 mm versus 2.533 ± 0.076 mm for the ACA VDom and MCA VDom, respectively.

**FIGURE 7:**
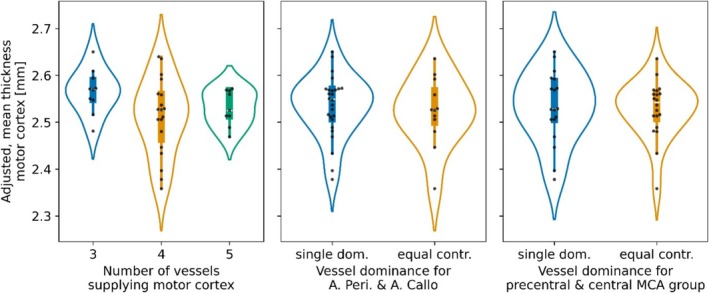
Effect of vessel patterns and dominances on mean thickness of the motor cortex. Group comparisons are shown as box plots with swarm and violin plots overlaid. No test returned a significant group difference.

For visual comparison, the covariate‐adjusted cortical thickness estimates are group‐wise plotted for vessel patterns, ACA VDom, and MCA VDom in Figure 7, respectively.

Analogously to the VDom ratings with equidistant thresholds, using thresholds derived in accordance with Ugur et al. showed no significant differences in cortical thickness in relation to ACA and MCA VDom ratings, respectively (see Figure S12). However, the MCA VDom rating with thresholds in accordance with Ugur et al. showed lower *p* values for the MCA VDom coefficient in the full model (*p* = 0.054) as well as the ANOVA model comparison (*p* = 0.130).

Additional analysis of vessel patterns can be found in the Supporting Information, that is a comparison of inter‐hemispheric differences in vessel patterns versus cortical thickness and assessment of vessel radii versus vessel pattern. Although elucidating the relation of vasculature and structure further, no statistical test performed reached significance.

To conclude, the addition of the vasculature to predict cortical thicknesses in a cohort of young, healthy volunteers: (i) can cause significant coefficients within the model, but the model itself never reaches significance, (ii) can increase the adjusted *R* ^2^ values compared to a covariate‐only model, and (ii) does not improve the predictive power compared to a covariate‐only model.

## 4 Discussion

We combined high‐resolution, motion‐corrected 7 T MRI with a novel, VDM‐based image postprocessing to enable the first in vivo assessment of the motor cortex vessel patterns and dominances. Previous post mortem descriptions were exceeded by providing complementary vessel pattern and vessel dominance estimates. For this holistic and data‐driven assessment of the vasculature, the proximity of a voxel to the vessels of interest was used to compute vessel‐specific supply volumes. Further, this approach enabled us to generate 3D vessel pattern‐specific supply territory atlases and assess the potential effect of the vascularization on the cortical thickness of the motor cortex.

Although considerable variance in the vascularization existed in the 38 hemispheres studied here, distinct vessel patterns could be identified and ratios of vessel dominance estimated. The number of vessels contributing to the motor cortex supply ranged from 3 to 5 vessels, with the 4‐vessel pattern being observed in 50% of all cases. Although the precentral and central groups of the MCA were always identified, the postcentral group was found in 11 out of 38 hemispheres, with highly variable supply volume fractions. For the ACA branches, which exclusively supplied the paracentral gyrus, the A pericallosa and A. callosomarginalis were found in 34 and 30 hemispheres, respectively. Although for the ACA there was an approximately equal amount of A. pericallosa dominance, A. callosomarginalis dominance, and equal contribution, for the MCA, the precentral and central group had equal contribution in approximately 50% of all cases.

Motivated by the distinctively different vessel configurations, that is the absence or presence of vessels, vessel pattern‐specific supply atlases were constructed in MNI space. To our best knowledge, this is the first attempt to provide 3D models of the motor cortex vascularization beyond a single normative atlas. Overall, the atlases showed stable borders across patterns for the regions assigned to the ACA branches and MCA groups (approx. 40% vs. 60% split of the motor cortex volume). Therefore, the ACA‐MCA‐watershed region might be stable across patterns, but within the parents' territory, inter‐individual variations exist. In the absence of an ACA branch, that is A. pericallosa and A. callosomarginalis, its counterpart supplied the region otherwise associated with the missing vessel. For the MCA postcentral group, the contribution was arguably neglectable for 5‐vessel patterns (9 out of 38 cases) and only for two hemispheres with four vessels supplying, the postcentral group mimicked the central group's supply, with the latter one being almost nonpresent in the pattern. Although the vessel supply regions of the parent vessels, that is ACA and MCA, can be interpreted as the superposition of their respective branches and groups, a single normative atlas or average would not be able to capture the differences observed across the six distinct vessel patterns truthfully.

Despite inter‐individual differences in motor cortex vascularization, the addition of the vasculature to OLS regression models did not significantly improve cortical thickness prediction beyond a covariate‐only model in this healthy young cohort. However, configurations with fewer supplying vessels showed slightly greater cortical thickness and larger radii, echoing findings by Gutierrez et al. who reported that thicker Circle of Willis arteries (e.g., MCA, ACA) correlate with better cognition (Gutierrez et al. 2018). Note that these results do not contradict the potential vascularization‐induced reserve mechanism for pathologies, as seen in hippocampal vessel patterns of CSVD patients (Perosa et al. 2020; Vockert et al. 2021; Garcia‐Garcia et al. 2023). The motor cortex typically exhibits minimal variation in cortical thickness and is generally free from pathological alterations at this young age, rendering compensatory vascular mechanisms dormant.

The validity of the method and results is of utmost importance. Motion‐corrected, 7 T MPRAGE images at 0.45 mm isotropic resolution were used. The improved vessel depiction of MPRAGE at 7 T compared to MPRAGE at lower field strengths and 3 T Time‐of‐Flight angiography (Maderwald et al. 2008; Wrede et al. 2014; Madai et al. 2015) in addition to the high resolution used and motion correction applied, enabled the identification of the vessels of interest. As vessel delineation and motor cortex segmentation were performed with the same data, vascularization and structure are inherently aligned, preventing any bias due to co‐registration steps. Further, motion correction increased the image quality for MPRAGE data used here (shown previously (Sciarra et al. 2022)) and improves vessel depiction at 7 T in general (Mattern et al. 2018, 2019). In the future, time‐of‐flight angiography at even higher resolutions could depict the pial arteries of the motor cortex (Bollmann et al. 2022). For follow‐up studies, employing (semi‐)automatic vessel segmentation instead of fully manual delineation is recommended. Although our manual delineation achieved good inter‐rater agreement, such an approach would further mitigate rater fatigue and considerably speed up the segmentation process.

Although the vessels directly perfusing the cortex—that is the capillary network fed by arterioles—are below the resolution limit of this study, the larger upstream vessels investigated in this study are an integral part of the supply network, and past studies showed their relation to cortical thickness and cognition: Bernier et al. used 3 T angiography and venography at lower resolutions and found significant correlation of vessel density to cortical thickness across brain regions (Bernier et al. 2018), and Vockert et al. found that hippocampal vessel patterns were associated with hippocampal and total gray matter volume in elderly participants (Vockert et al. 2021).

Supply territories were estimated with a VDM‐based approach with similarity to another data‐driven estimation of vascular territories (Mut et al. 2014). Although VDM was successfully applied to study vascular reserve mechanisms in CSVD patients (Garcia‐Garcia et al. 2023), it is no perfusion measurement. Nevertheless, the assumption that the vessel‐voxel‐distance correlates with the probability that the voxel is supplied by that vessel is reasonable (Feekes 2006; Feekes et al. 2005; Haast et al. 2024) and post mortem studies of the motor cortex employed no perfusion measurements either, but are based on the visual expert assessment of the vasculature around the ROI (Ugur et al. 2005; Frigeri et al. 2015). Hence, the VDM‐based approach mimics to some extent the expert assessment in post mortem studies. Further, our approach can estimate supply volumes, both relative and absolute, which could not be generated from visual assessment. Our approach follows a “winner takes all” idea, that is regardless of how close two vessels are to a certain voxel only the closest vessel is considered and no overlap of supply regions is estimated. The subsequently generated 3D supply atlases resemble supply territories typically found in textbooks, but are not based on vessel‐specific perfusion measurements. Obtaining these ground truth vessel‐specific perfusion measurements would require either perfusing the brain post mortem with a tracer or performing vessel territory imaging in vivo (Feekes 2006; Hartkamp et al. 2013; Zhang et al. 2021) which is nontrivial given the number of relevant vessels, their close proximity and small size. Although we used Euclidean distances for their reproducibility and prior success in related work, a geodesic distance metric may offer a complementary perspective by more closely following vascular branching paths—especially as imaging resolution improves.

Although further studies are required to validate the generalizability of the vessel patterns and dominances found here, our sample size is comparable to the post mortem study by Ugur et al. (38 vs. 40 hemispheres) (Ugur et al. 2005). Further, our results are in large part in accordance with the post mortem results with the exception of the central group dominance. Given the high agreement between Ugur et al. and our ACA VDom estimates and the robustness towards the thresholds used for the ACA VDom, that is near‐identical performance of the equidistant thresholds and the optimized thresholds, a systematic bias in the assessment of the MCA central group's contributions to the supply of the motor cortex due to methodological differences between the studies and the complex folding of the precentral gyrus caused the differences in MCA VDom frequencies. Our good inter‐rater agreement and adherence to the vessel description provided by Ugur et al. render a systematic bias due to the rater or vessel identification less likely. Most likely, the observed discrepancy can be attributed to methodological differences, that is resolution limited, quantitative volume versus invasive, qualitative surface area estimates. Our 7 T MRI approach, whereas resolution‐limited, provided noninvasive assessment within the motor cortex from arbitrary orientations without tissue sectioning. This enabled quantitative, volumetric assessment of vessel dominance, contrasting the qualitative, visual assessment by Ugur et al. which likely focused on the surface area of the motor cortex near each vessel group. Given the fact that the precentral gyrus (mainly supplied by MCA groups) is a more convoluted structure compared to the paracentral gyrus (supplied by the ACA branches), differences in quantitative volume estimates versus perceived supplied surface areas may have contributed to the observed discrepancy. Furthermore, Ugur et al. qualitative visual dominance ratings are arguably more subjective and nontrivial to reproduce across raters than our quantitative approach, which included rigorous threshold testing. Crucially, neither method leveraged true perfusion measurements, and thus a definitive ground truth for vessel dominance remains elusive. Despite these methodological differences, the overall concordance between post mortem and in vivo results was encouraging, that is agreement of ACA VDom rating prevalence, precentral group dominance, absence of postcentral group dominance, and few exclusion cases with bilateral supply from a single ACA branch. Hence, the comparison to Ugur et al. indicates that our noninvasive, in vivo, and translationally applicable approach can produce results similar to post mortem expert ratings. Beyond translating vessel pattern and dominance assessment from post mortem to in vivo, to our best knowledge for the first time, we developed tools to compute 3D supply territory atlases. Much like the hippocampal work by Spallazzi et al. (Spallazzi et al. 2018), which laid the groundwork for translation to CSVD, these methods enable longitudinal assessment of the vascularization, for example to study vascular reserve in aging or ALS patients (Schreiber et al. 2023). Furthermore, the approach can be translated from the motor cortex to other brain regions to establish vessel patterns throughout the brain, potentially challenging the use of single normative descriptions of the vasculature.

## 5 Conclusion

To summarize, we presented a successful translation of motor cortex vessel assessment from post mortem to in vivo using 7 T MRI and VDM. We provided a holistic description of the motor cortex vessel patterns and dominances to study vascular reserve in disease and health in the future.

## Conflicts of Interest

The authors declare no conflicts of interest.

## Supporting information


**Data S1:** hbm70311‐sup‐0001‐DataS1.

## Data Availability

According to the IRB data privacy agreement, we are restricted to share any subject‐specific data. However, the here presented vessel pattern atlases in MNI space (without any subject‐specific information) are publicly available https://doi.org/10.5281/zenodo.15673561 (Mattern et al. 2025). The VDM toolbox is public, as referred to in the manuscript.
