# Supplementary material for: Assessing Arterial Patterns in the Motor Cortex With 7 Tesla Magnetic Resonance Imaging and Vessel Distance Mapping
Source: Hum Brain Mapp. 2025 Aug 5;46(11):e70311. doi: 10.1002/hbm.70311 (PMC12322923; doi:10.1002/hbm.70311)
Supplement: Supplementary file 1 — Data S1: hbm70311‐sup‐0001‐DataS1. [file HBM-46-e70311-s001.docx]

# **Supplementary material**

## **Delineation of arterial vasculature**

The ACA was segmented from the pars praecallosa on. The three groups of the MCA were segmented as soon as they were in the proximity of the motor cortex (see Fig. S1).

By using the zoom-function in Mango the vessels were followed from their thick beginning to thin end and also vice versa to see if they reach the primary motor cortex in sagittal view. If the view from sagittal was not clear the perspective was changed to a coronal or transversal view. The segmentation was made in sagittal view. After that it was corrected in coronal view. Vessels with a maximum of three voxels distance from the primary motor cortex were still considered, hence delineated, due to their spatial vicinity.

The identification of vessel branches and groups followed the explanation provided by Ugur et al. as well as anatomical atlases^1^. For the identification of the callosomarginal artery it is decisive that the vessel has a certain thickness and prominence and that it runs through the sulcus cinguli. The criterion for the identification of the pericallosal artery is a course that at least at the beginning runs by the corpus callosum. For the MCA groups the precentral and central group were differentiated by checking if most of the arteries were running through the precentral sulcus and entered the motor cortex rostrally, i.e. precentral group, or if the majority of vessels propagated through the central sulcus and entered the motor cortex caudally, i.e. central group. For the postcentral group, the majority of vessels were traversing through the postcentral sulcus towards the primary motoric cortex.

*
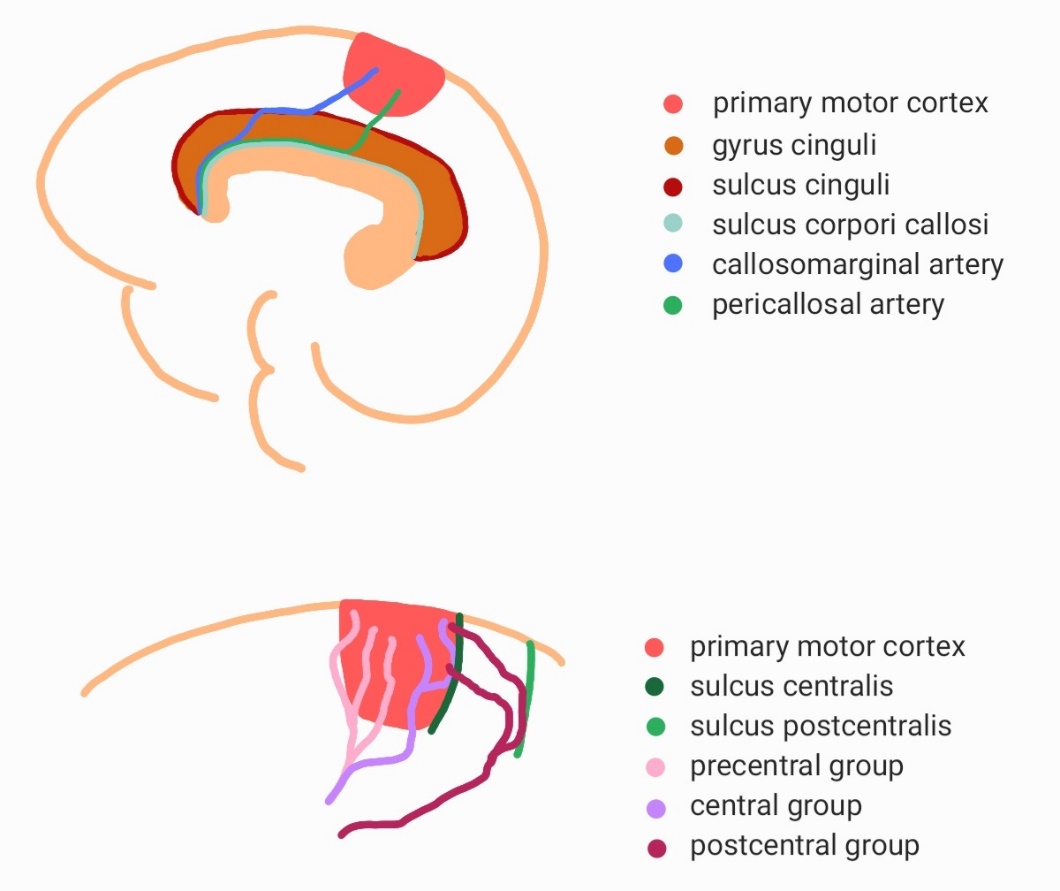

Figure S1: Schematic sagittal views of the arteries supplying the motor cortex.*

## **Vessel-specific average distances in MNI space**


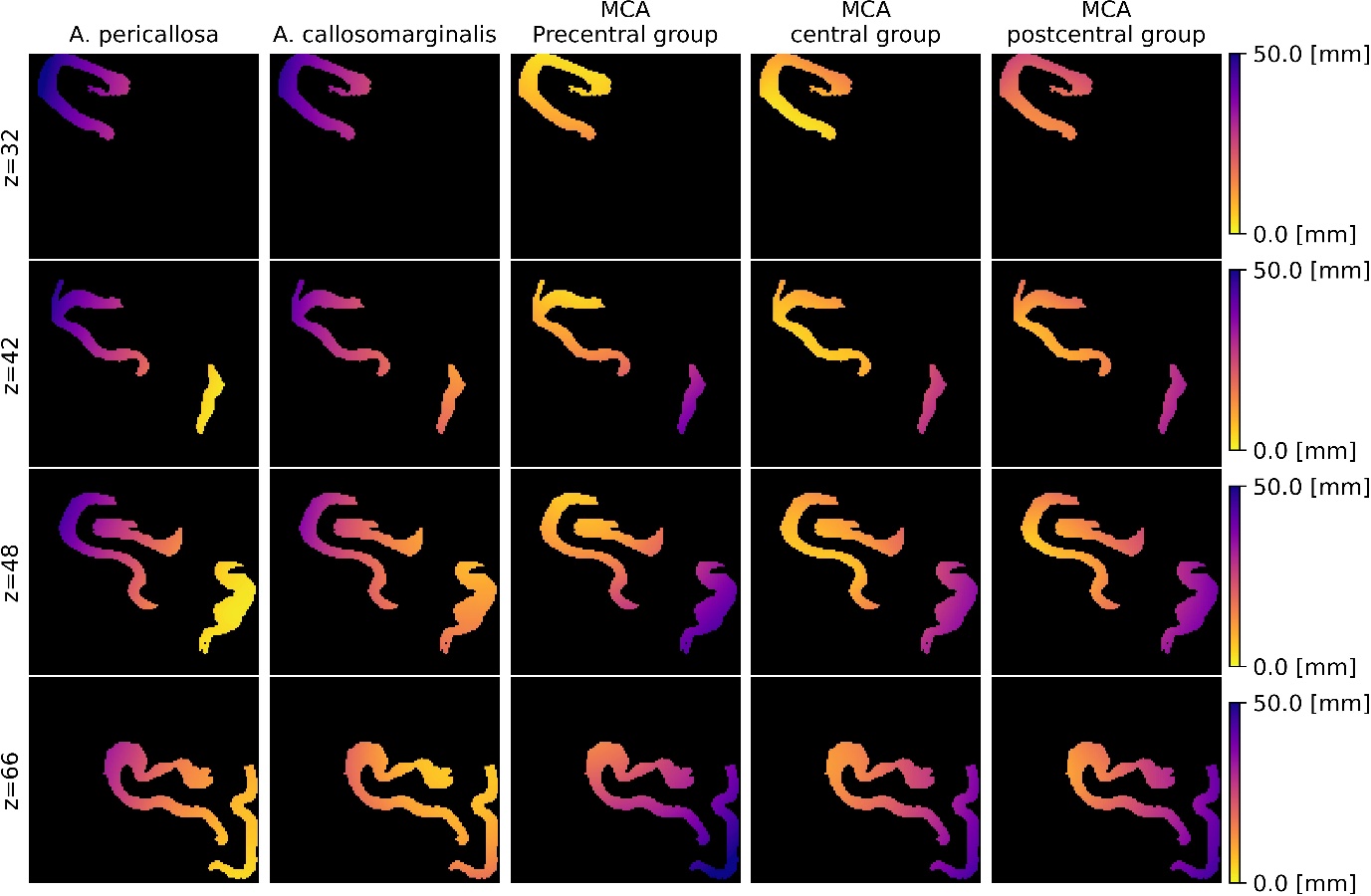


*Figure S2: Comparison of average distance maps per artery in MNI space.*

For each motor cortex voxel, the average distance to all arteries of interest were computed (per hemisphere in native space) and, subsequently, transformed into MNI space (right hemisphere flipped and co-registered to left hemisphere of MNI template). Representative axial slices cropped to the left motor cortex (see Fig. S2, location in MNI space indicated by z-position) show distinct differences in the vessels’ trajectories. While the ACA vessels, i.e. A. pericallosa and A. callosomarginalis return the shortest distances for the medial part of the motor cortex, MCA arteries show lower distances more laterally. Note that low distances are indicated by bright colors, i.e. voxels being close to the artery of interest.

## **Bifurcation and subbranches analysis**

In addition to vessel pattern and dominances, we performed an analysis of the bifurcations to provide additional insights on the motor cortex vasculature.

For the ACA branches, a total of 73 subbranches in 34 hemispheres (on average 2.147 ± 1.184 subbranches per hemisphere) and 74 subbranches in 30 hemispheres (on average 2.467 ± 1.332 subbranches per hemisphere) were found for the A. pericallosa and the A. callosomarginalis, respectively. For the MCA groups, a total of 76 subbranches in 38 hemispheres (on average 2.000 ± 1.040 subbranches per hemisphere), 81 subbranches in 38 hemispheres (on average 2.132 ± 0.963 subbranches per hemisphere), and 11 subbranches in 11 hemispheres (on average 1.000 ± 0.000 subbranches per hemisphere) were found for the precentral, central, and postcentral group, respectively. Further, the relative frequencies for each bifurcation level has shown in Fig. S3.

While the number of subbranches for the A. pericallosa and A. callosomarginalis are approximately the same, the subbranches of the A. callosomarginalis supplying the motor cortex tend to branch off at higher bifurcation levels than the subbranches of the A. pericallosa. The precentral and central group of the MCA have very similar bifurcation patterns and, in line with the ACA branches, have approximately 2 subbranches per hemisphere going to the motor cortex. In line with the vessel pattern, the postcentral group is an exception. If present, only a single subbranch of the postcentral group reached the motor cortex. Further, this subbranch most likely occurs at the sixth or seventh bifurcation level, while all other subbranches tend to branch off between the second and fifth bifurcation level


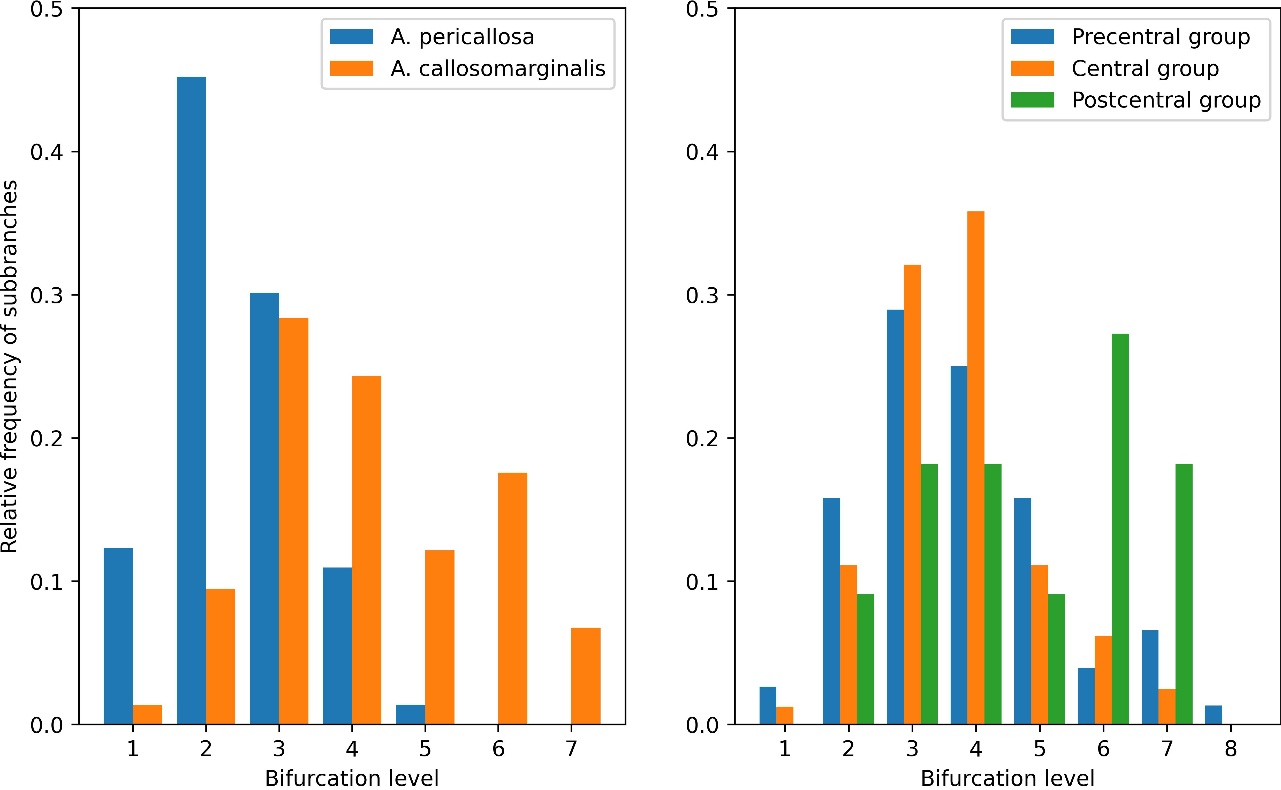


*Figure S3: Comparison of the relative frequencies of subbranches per bifurcation level. For the ACA branches, the A. pericallosa branches off at lower bifurcation levels than the A. callosomarginalis. For the MCA groups, the Postcentral group has considerably high bifurcations levels (6-7).*

## **Supply volume fractions per hemisphere**


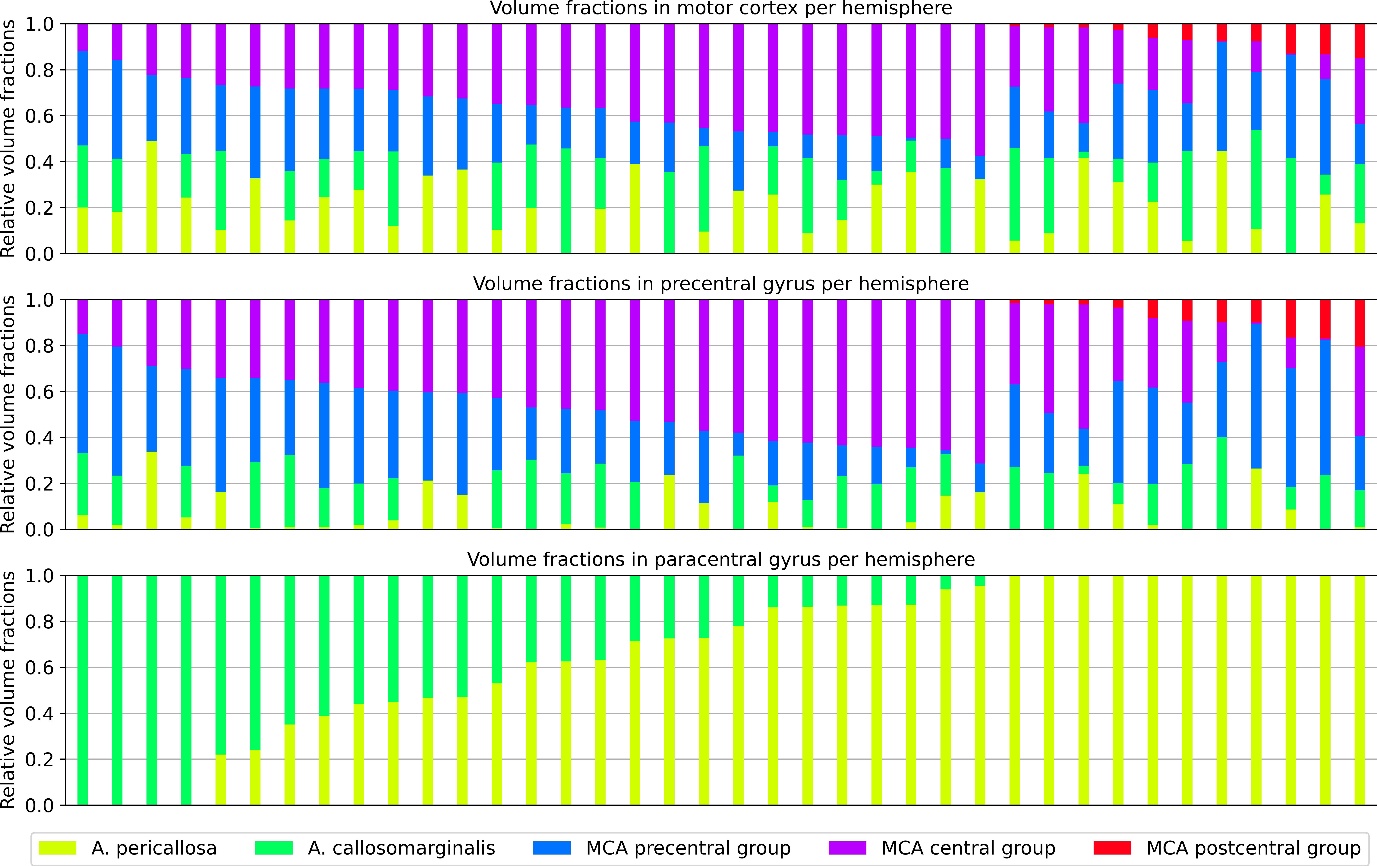


*Figure S4: Per hemisphere the supply volume fraction for all arteries are shown for the motor cortex, precentral gyrus, and paracentral gyrus, respectively.*

Comparing the VDM-based supply volume across hemisphere yields considerable inter-hemisphere variability (see Fig. S4). The estimates for the motor cortex are shown along its subregions, i.e. the precentral and paracentral gyrus, respectively. While the entire motor cortex and its subregion, the precentral gyrus, are supplied by up to five different vessels, the paracentral gyrus is exclusively supplied by the ACA-vessels, A. pericallosa and A. callosomarginalis.

In addition to the Fig. S4. with all hemispheres pooled together, a hemisphere-wise comparison of the supply volumes is provided in Fig. S5.


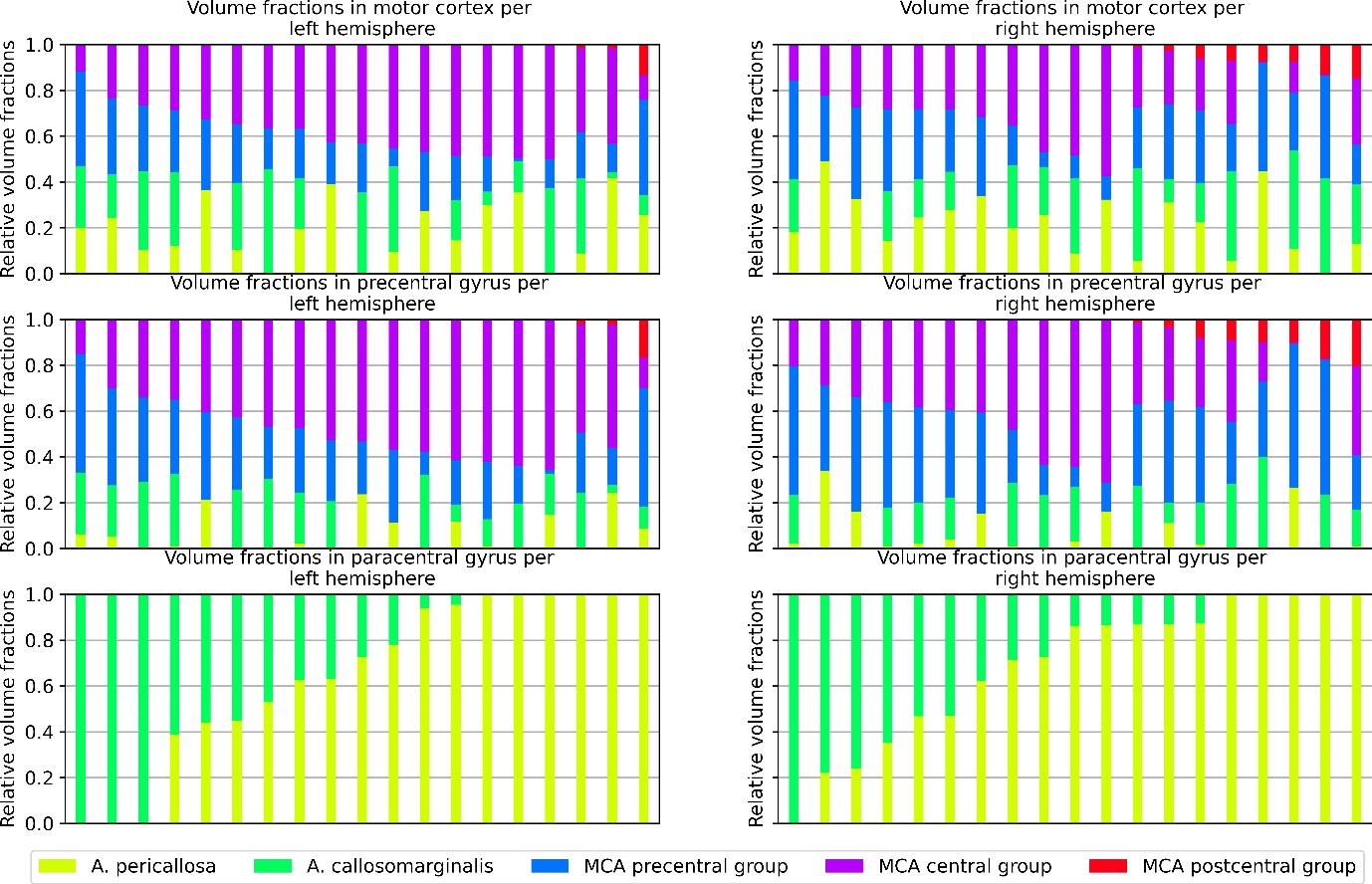


*Figure S5: The supply volume fraction for all arteries are shown for the motor cortex, precentral gyrus, and paracentral gyrus for the left and right hemisphere, respectively.*

Note that the hemispheres were ordered with the following logic: For the motor cortex and precentral gyrus, the primary sorting criterion was the contribution of the post-central group, followed by the central group's contribution, and finally by the contribution of the A. pericallosa. This approach aimed to meaningfully separate the post-central contribution, which was present in only 11 out of 38 hemispheres, from the subsequent contributions of the precentral group and A. pericallosa. For the paracentral gyrus, the data was sorted solely by the contribution of the A. pericallosa.

## **Rater agreement**


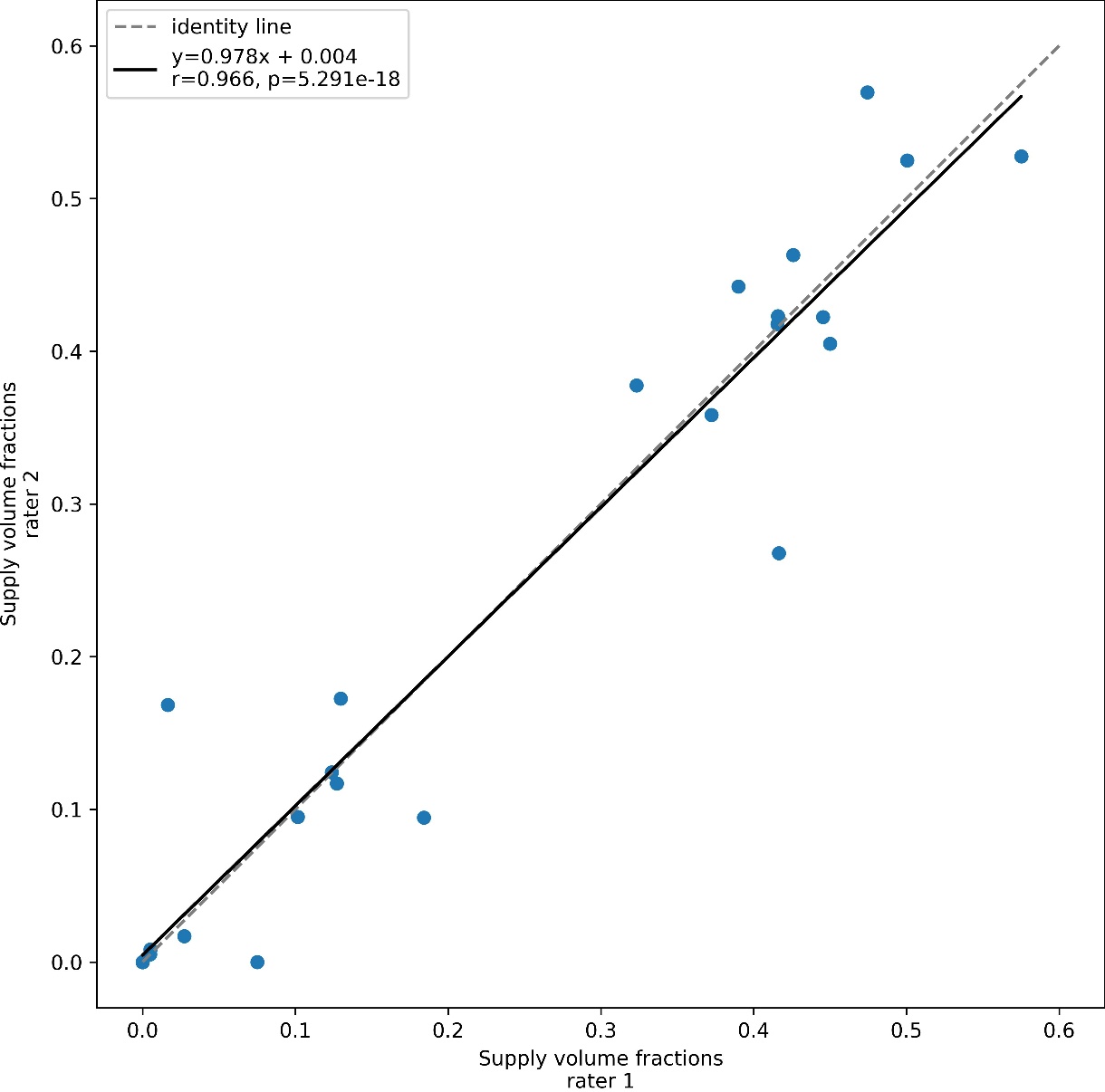


*Figure S6: Comparison of volume fractions computed from vessel delineations from two different raters for six hemispheres (plotted as dots). The Pearson correlation of volume fractions is high and significant. Further, the slope and offset of the regression line fitted (solid black line) is close to the identity line (dashed gray line).*

To assess a potential rater bias in our vessel pattern and dominance analysis, six representative hemispheres (i.e. each hemisphere with a different vessel configuration) the vessels of interest were delineated by a second rater. Based on the second rater’s segmentations, the supply volume fractions were estimated via VDM as described in the method section. The comparison of both raters (see Fig. S6) yields a high and significant Pearson correlation (r=0.966, p<0.001) and the linear regression returns slope and offset close to the identity line (slope=0.978, offset=0.004). To conclude, overall an excellent rater agreement of supply volume fractions was observed for the representative subset of our data, albeit volume fractions of a few hemispheres show deviations from a pure linear behavior.

## **Prevalence of vessel patterns for the paracentral and precentral gyrus**


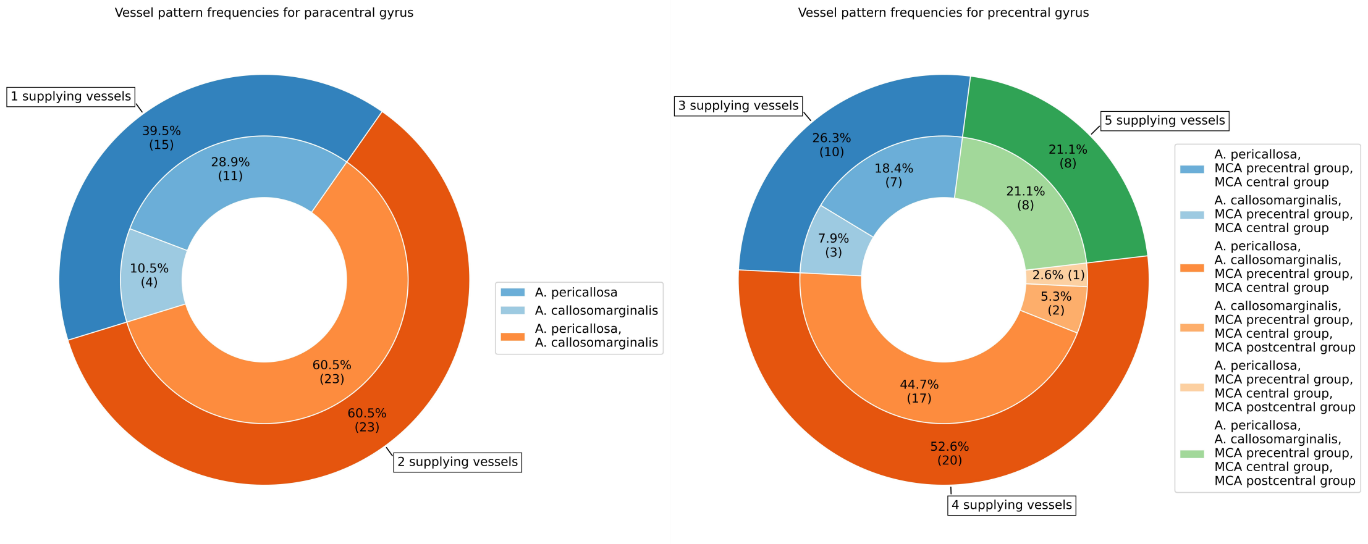


*Figure S7: Prevalence of vessel supply patterns for the paracentral and precentral gyrus as estimated with VDM.*

As a complementary assessment, the vessel patterns were analyzed for the paracentral and precentral gyrus, respectively (see Fig. S7). In the paracentral gyrus, only the ACA vessels A. pericallosa and A. callosomarginalis contribute to the VDM-based supply. In 60.5% of all hemisphere both vessels supplied the paracentral gyrus while in remaining hemispheres only one of the two arteries supplied (28.9% and 10.5% for the A. pericallosa and A. callosomarginalis, respectively). In the precentral gyrus, more diverse supply patterns can be observed. In line with the results of the motor cortex, the supply was provided by three, four, or five vessels with a prevalence of 26.3%, 52.6%, and 21.1%, respectively. While approx. half all hemispheres are supplied by four vessels, out of these 20 cases, 17 had a vessel configuration involving both ACA vessels and the MCA precentral and central group. The remaining three hemispheres had all MCA groups and one of the ACA vessels supplying the precentral gyrus. Similarly, for 10 hemispheres supplied by three vessels, the supplying vessels where the MCA precentral and central group along with one of the ACA vessels (7 and 3 cases for A. pericallosa and A. callosomarginalis, respectively). The remaining eight hemispheres were supplied by all five vessels.

## **MNI supply volume fractions and average ratios**

The supply volume fractions in MNI space for the motor cortex per vessel pattern are shown in Tab. S1, followed by the average volume ratios for the motor cortex, precentral gyrus, and paracentral gyrus in Tab. S2.

*Table S1: Supply volume fraction in MNI space per vessel pattern*

| Pattern name | Pattern frequency | Avg. volume fraction  A. pericallosa | Avg. volume fraction  A. callosomarginalis | Avg. volume fraction  MCA precentral | Avg. volume fraction  MCA central | Avg. volume fraction  MCA postcentral |
| --- | --- | --- | --- | --- | --- | --- |
| 3 supplying vessels (A. callo. variant) | 3 | 0.000 | 0.416 | 0.155 | 0.429 | 0.000 |
| 3 supplying vessels (A. peri. variant) | 7 | 0.406 | 0.000 | 0.225 | 0.369 | 0.000 |
| 4 supplying vessels (ACA double variant) | 17 | 0.185 | 0.259 | 0.184 | 0.372 | 0.000 |
| 4 supplying vessels (A. callo. variant) | 1 | 0.000 | 0.415 | 0.394 | 0.012 | 0.179 |
| 4 supplying vessels (A. peri. variant) | 1 | 0.450 | 0.000 | 0.461 | 0.005 | 0.084 |
| 5 supplying vessels | 9 | 0.153 | 0.285 | 0.243 | 0.296 | 0.023 |

*Table S2: Supply volume ratios for motor cortex, precentral gyrus, and paracentral gyrus, respectively*

|  | Motor cortex | Precentral gyrus | Paracentral gyrus |
| --- | --- | --- | --- |
| Ratio of ACA to entire supply | 0.411±0.057 | 0.240±0.064 | 1.000±0.000 |
| Ratio of pericallosa to entire ACA supply | 0.518±0.344 | 0.324±0.404 | 0.674±0.0334 |
| Ratio of central group to MCA supply (excluding postcentral group) | 0.550±0.225 | 0.550±0.225 | 0.000±0.000 |
| Ratio of postcentral group to MCA supply | 0.035±0.070 | 0.035±0.070 | 0.000±0.000 |

## **Axial views of vessel patterns in MNI space**


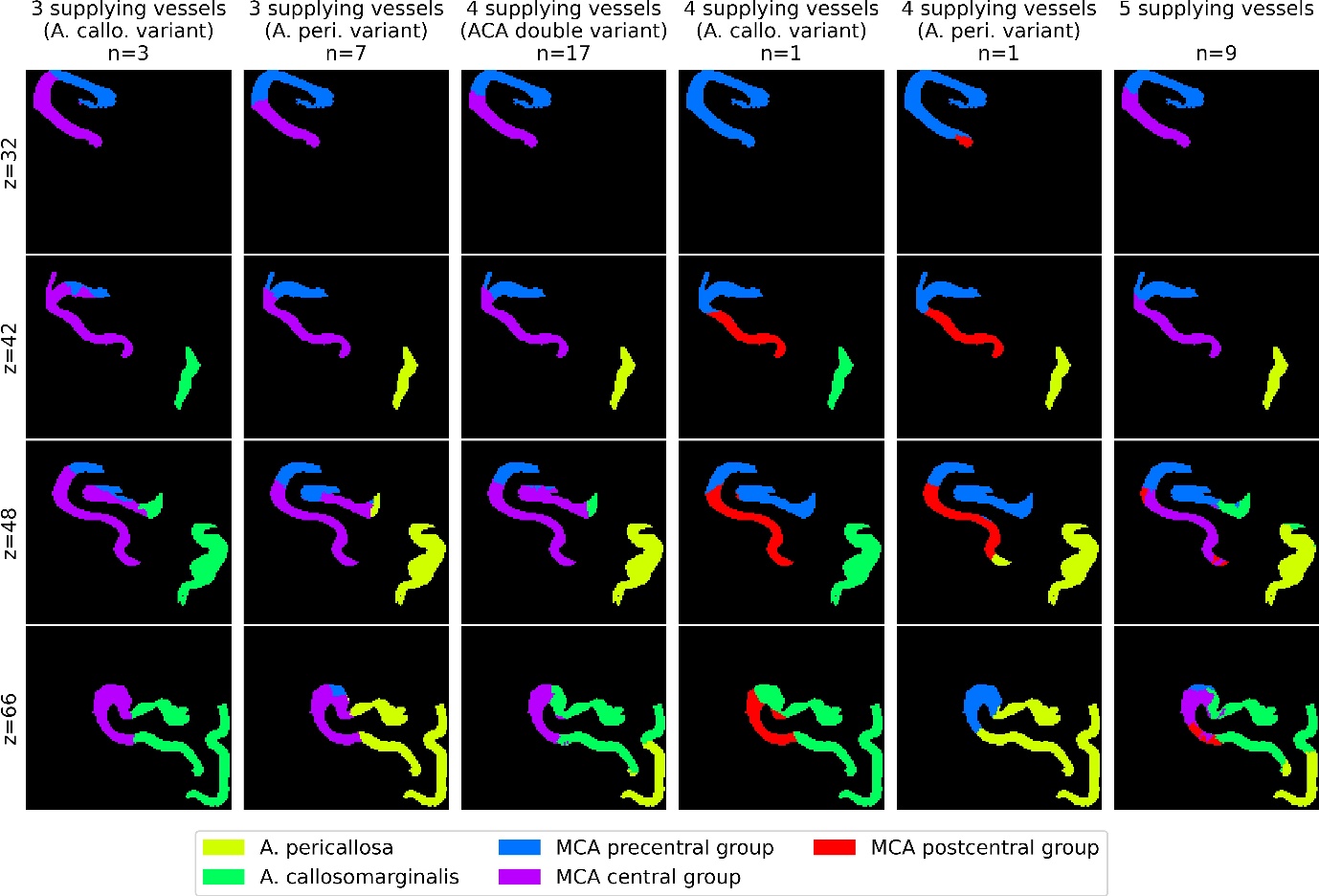


*Figure S8: Representative axial slices in MNI space of the six vessel patterns (slice position in MNI space indicated on the left). After co-registering all hemispheres to the same space, for each voxel the most frequent artery label, i.e. majority vote, was assigned.*

## **Effect of threshold selection on vessel dominance assessment**

To compare the qualitative vessel dominance (VDom) ratings by Ugur et al.^1^ with our continuous volume ratios, we applied a lower and upper threshold to categorize our VDom estimates. If a volume ratio was between the lower and upper threshold, the contribution of both vessel of interest was consider to be equal and otherwise one vessel would be dominant.

To estimate the effect of the thresholds von VDom and find the thresholds returning the highest agreement with Ugur et al.^1^ the following steps were performed:

First, 100 pairs of lower and upper thresholds were generated by computing equally spaced lower thresholds in the interval of 0.05 to 0.45 and computing the respective upper threshold as 1 minus the lower threshold (corresponding to the range of 0.95 to 0.55). Therefore, the threshold pair of 0.05 and 0.95 would be the most exclusive choice for single vessel dominance as any volume ratio between 0.05 and 0.95 would be considered equal contribution of both vessels. The other extreme would be the threshold pair of 0.45 and 0.55, rendering only volume ratios approx. 50:50 as equal contribution. The threshold pair of 0.33 and 0.66 would be consider as equally spaced and is used in the main manuscript to determine VDom frequencies.

Second, to quantify the agreement with Ugur et al^1^, the cumulative absolute difference was computed. Per threshold pair, the VDom frequencies in percent were subtracted from the corresponding frequencies reported by Ugur et al^1^ and their absolute sum is reported.

The corresponding results for the ACA branches and MCA vessel groups are shown in Fig. S9, respectively. Additionally, Tab. S3, shows the thresholds returning highest agreement with Ugur et al. for ACA and MCA vessels, respectively.

On the left in Fig. S9 the volume ratios for all hemispheres are shown. To compute the VDom frequencies, a lower and upper threshold are utilized and the number of hemispheres, below, in between, and above the thresholds are counted. By simultaneously increasing the lower and decreasing the upper threshold, the criteria for equal distribution gets more and more restrictive and, therefore, the associated frequency of equal contribution is decreasing (see Fig. S9, right column). Analogously, the frequencies for single vessel dominance vary while the sum of all three VDom frequencies always return 100%. In addition, the cumulative absolute difference w.r.t. Ugur et al.^1^ is reported (grey dashed line, separate y-axis on the right).


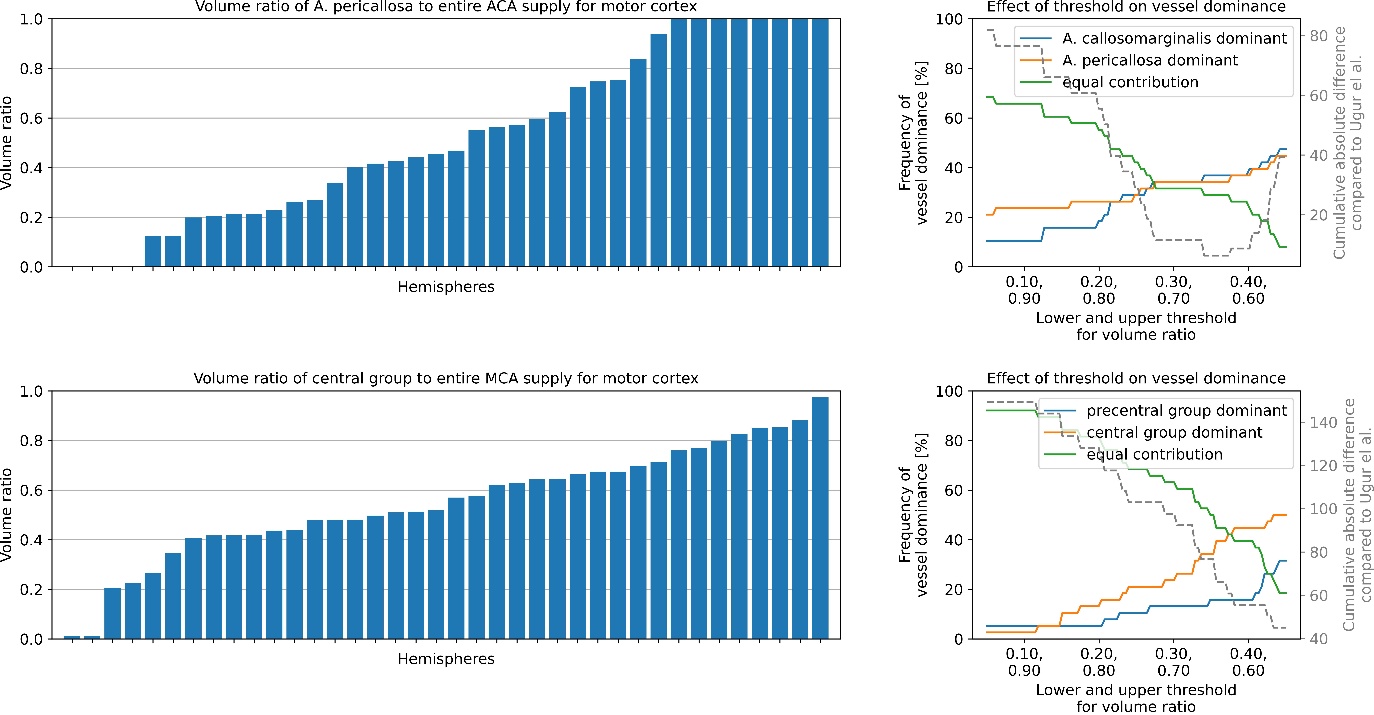


*Figure S9: Volume ratios for the ACA branches and MCA groups are shown on the left. By applying the lower and upper threshold to these volume ratios, vessel dominance frequencies can be obtained. These frequencies and their respective cumulative absolute difference to Ugur et al. for varying threshold pairs are shown on the right.*

For the VDom of the ACA branches, the optimal lower and upper thresholds (cumulative absolute difference to Ugur et al.^1^ was 6.316) are 0.340 and 0.660, respectively, virtually the same as the equally spaces thresholds used in the main manuscript. Further, the cumulative absolute difference to Ugur et al.^1^ is arguably low in the thresholds from 0.300 and 0.700 to 0.400 and 0.600, suggesting robustness of the VDom frequencies to variations of thresholds used.

In contrast, for the precentral and central group of the MCA, the agreement with Ugur et al. is rather poor. Even with the optimized thresholds, a cumulative absolute difference of 45% is present. Further, the trend of the cumulative absolute difference (see Fig. S9) suggests that for improved agreement with Ugur et al.^1^, the lower threshold should be higher than 0.5. This stems from the fact that Ugur et al.^1^ reported a dominance of the central group in 72.5% of the cases, while with our data it reached 50% maximum. Given the excellent agreement for the ACA branches, this suggest a systematically different rating of the central group in Ugur et al. and our study.

*Table S3: Thresholds optimized w.r.t. Ugur et al. and their respective cumulative absolute difference for the vessel dominance (VDom) for the ACA branches (A. callosomarginalis vs A. pericallosa) and MCA groups (central vs. precentral group)*

|  | VDom of ACA branches | VDom of MCA groups |
| --- | --- | --- |
| optimal lower threshold | 0.340 | 0.434 |
| optimal upper threshold | 0.660 | 0.566 |
| cum. abs. difference for optimal threshold set | 6.316 % | 45.000 % |

To additionally support these findings, the VDom frequencies for the equidistant and optimized thresholds are compared (see Fig. 6 and Fig. S10). The ACA-branches VDom frequencies are A. pericallosa dominant 34.2% vs 34.2%, A. callosomarginalis dominant 34.2% vs 36.8%, and equal contribution 31.6% vs 28.9% for equidistant vs. Ugur et al. optimized thresholds, respectively. The MCA group VDom frequencies are central group dominant 34.2% vs 50.0%, precentral group dominant 13.2% vs. 26.3%, and equal contribution 52.6% vs. 23.7% for equidistant vs. Ugur et al. optimized thresholds, respectively.


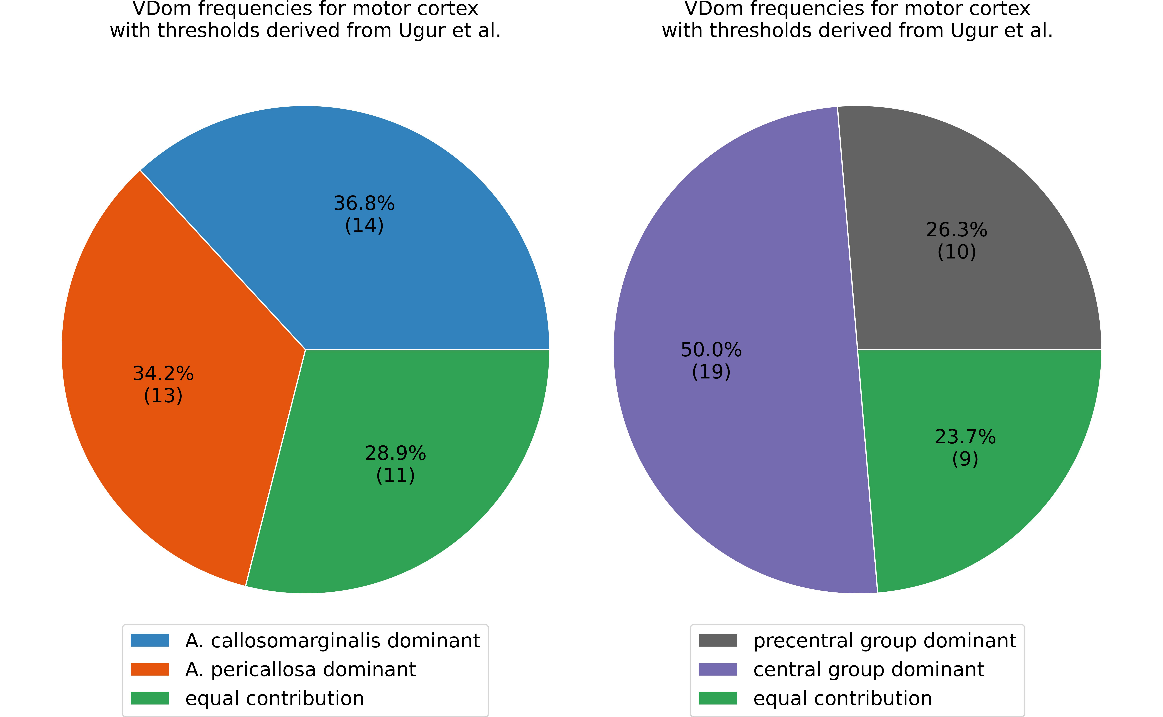


*Figure S10: Vessel dominance (VDom) using thresholds optimized for highest agreement with Ugur et al. for the ACA branches, i.e. A. callosomarginalis and A. pericallosa, and MCA groups, i.e. precentral and central group.*

## **Statistical assessment of covariates on cortical thickness estimates of the motor cortex**


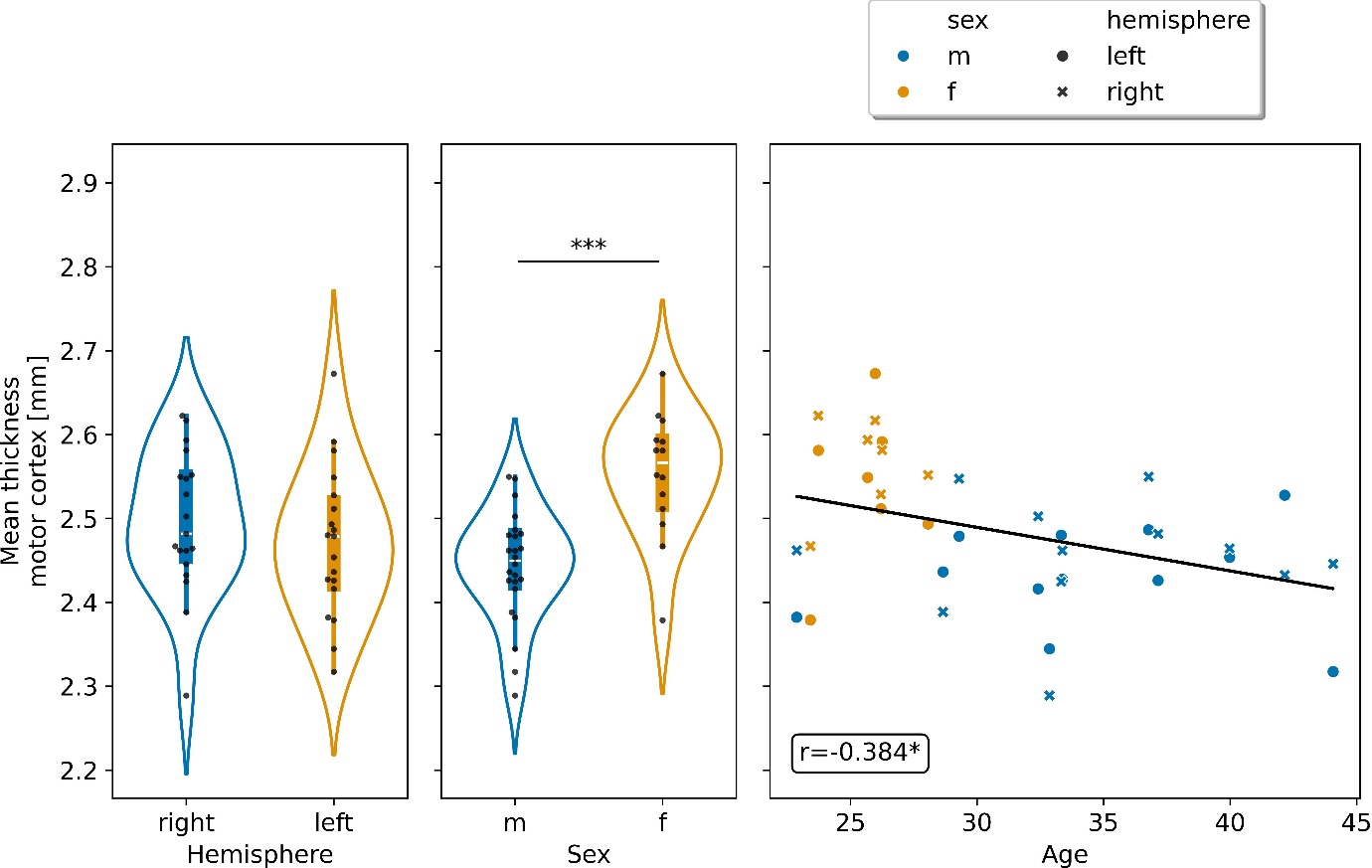


*Figure S11: Effect of covariates on mean thickness of the motor cortex. Categorial independent variables are shown as group comparisons with overlaid box, swarm, and violin plots while continuous variables are shown with as scatter plots with the results of the linear regression overlaid. Significance is indicated as * for p<0.05, ** for p<0.005, and *** for p<0.0005.*

The cortical thickness of the left and right hemisphere were 2.471±0.087 mm and 2.495±0.082 mm, respectively, and showed no significant difference [t(36) = -0.854 , p = 0.399, d = 0.277] (see Fig. S11). Cortical thickness between males and females were significantly different [t(24.561) = -4.607 , p < 0.001, d = 1.604] with average thicknesses of 2.443±0.064 mm and 2.553±0.071 mm for males and females, respectively. Linear regression of age and cortical thickness showed a significant Pearson correlation [r(37) = -0.384, p = 0.035] with a slope of -0.005 mm per year and a y-axis offset of 2.645 mm. While the effect of age on cortical thickness was expected, the sex difference might be caused by the sampling bias in our cohort as the females included were among the youngest participants (females and males 25.6±1.5 and 34.4±5.8 years old, respectively).

## **Detailed statistics for vasculature vs. thickness**

Detailed test statistics for the analysis of vasculature vs. cortical thickness are provided in the Tables S4-S6 for vessel patterns, ACA dominance, and MCA dominance, respectively. For each analysis, we fitted a full model including the vascular metric alongside covariates, and a reduced model including only the covariates (age and sex). We then performed an ANOVA model comparison to assess whether the inclusion of the vascular variable significantly improved model fit. Note that the reduced model was identical across all comparisons, as it included only the covariates.

Table S4: Comparison of full and reduced model for vessel patterns, i.e. number of supplying vessels. The number of supplying vessels ranged from 3 to 5 and 3 was used as the reference among patterns.

| Full model: mean_thickness_motor ~ scan_age + sex + num_supplying_vessels | | | | | | |
| --- | --- | --- | --- | --- | --- | --- |
|  | Coef. | Std.Err. | z | P>\|z\| | [0.025 | 0.975] |
| Intercept | 2.585 | 0.073 | 35.265 | 2.023E-272 | 2.441 | 2.728 |
| sex[T.m] | -0.123 | 0.030 | -4.071 | 4.681E-5 | -0.182 | -0.064 |
| num_supplying_vessels[T.4] | -0.057 | 0.028 | -2.029 | 0.042 | -0.111 | -0.002 |
| num_supplying_vessels[T.5] | -0.037 | 0.022 | -1.663 | 0.096 | -0.081 | 0.007 |
| scan_age | 4.330E-4 | 0.002 | 0.178 | 0.859 | -0.004 | 0.005 |
| Reduced model: mean_thickness_motor ~ scan_age + sex | | | | | | |
|  | Coef. | Std.Err. | z | P>\|z\| | [0.025 | 0.975] |
| Intercept | 2.531 | 0.072 | 35.282 | 1.093E-272 | 2.390 | 2.671 |
| sex[T.m] | -0.118 | 0.030 | -3.916 | 9.005E-5 | -0.176 | -0.059 |
| scan_age | 0.001 | 0.003 | 0.335 | 0.738 | -0.004 | 0.006 |
| Model Comparison via ANOVA | | | | | | |
|  | df_resid | ssr | df_diff | ss_diff | F | Pr(>F) |
| Reduced Model | 35.000 | 0.169 | 0.000 |  |  |  |
| Full Model | 33.000 | 0.149 | 2.000 | 0.020 | 2.233 | 0.123 |

Table S5: Comparison of full and reduced model for ACA branch dominance, i.e. A. pericallosa and A. callosomarginalis. Dominance was either rated as single vessel dominance or equal contribution.

| Full model: mean_thickness_motor ~ scan_age + sex + dom_peri_callo | | | | | | |
| --- | --- | --- | --- | --- | --- | --- |
|  | Coef. | Std.Err. | z | P>\|z\| | [0.025 | 0.975] |
| Intercept | 2.529 | 0.072 | 35.043 | 4.99855116077687E-269 | 2.387 | 2.670 |
| sex[T.m] | -0.118 | 0.030 | -3.948 | 7.883E-5 | -0.177 | -0.059 |
| dom_peri_callo[T.single dom.] | 0.012 | 0.026 | 0.458 | 0.647 | -0.040 | 0.064 |
| scan_age | 0.001 | 0.003 | 0.261 | 0.794 | -0.004 | 0.006 |
| Reduced model: mean_thickness_motor ~ scan_age + sex | | | | | | |
|  | Coef. | Std.Err. | z | P>\|z\| | [0.025 | 0.975] |
| Intercept | 2.531 | 0.072 | 35.282 | 1.093E-272 | 2.390 | 2.671 |
| sex[T.m] | -0.118 | 0.030 | -3.916 | 9.005E-5 | -0.176 | -0.059 |
| scan_age | 0.001 | 0.003 | 0.335 | 0.738 | -0.004 | 0.006 |
| Model Comparison via ANOVA | | | | | | |
|  | df_resid | ssr | df_diff | ss_diff | F | Pr(>F) |
| Reduced Model | 35.000 | 0.169 | 0.000 |  |  |  |
| Full Model | 34.000 | 0.168 | 1.000 | 0.001 | 0.230 | 0.635 |

Table S6: Comparison of full and reduced model for MCA group dominance, i.e. central and precentral group. Dominance was either rated as single vessel dominance or equal contribution.

| Full model: mean_thickness_motor ~ scan_age + sex + dom_central_precentral | | | | | | |
| --- | --- | --- | --- | --- | --- | --- |
|  | Coef. | Std.Err. | z | P>\|z\| | [0.025 | 0.975] |
| Intercept | 2.529 | 0.078 | 32.383 | 4.698E-230 | 2.376 | 2.682 |
| sex[T.m] | -0.117 | 0.032 | -3.677 | 2.363E-4 | -0.179 | -0.054 |
| dom_central_precentral[T.single dom.] | 0.005 | 0.026 | 0.179 | 0.858 | -0.046 | 0.055 |
| scan_age | 0.001 | 0.003 | 0.318 | 0.751 | -0.004 | 0.006 |
| Reduced model: mean_thickness_motor ~ scan_age + sex | | | | | | |
|  | Coef. | Std.Err. | z | P>\|z\| | [0.025 | 0.975] |
| Intercept | 2.531 | 0.072 | 35.282 | 1.093E-272 | 2.390 | 2.671 |
| sex[T.m] | -0.118 | 0.030 | -3.916 | 9.005E-5 | -0.176 | -0.059 |
| scan_age | 0.001 | 0.003 | 0.335 | 0.738 | -0.004 | 0.006 |
| Model Comparison via ANOVA | | | | | | |
|  | df_resid | ssr | df_diff | ss_diff | F | Pr(>F) |
| Reduced Model | 35.000 | 0.169 | 0.000 |  |  |  |
| Full Model | 34.000 | 0.169 | 1.000 | 1.995E-4 | 0.040 | 0.842 |

## **Effect on vessel dominances derived from Ugur et al. on motor cortex thickness**


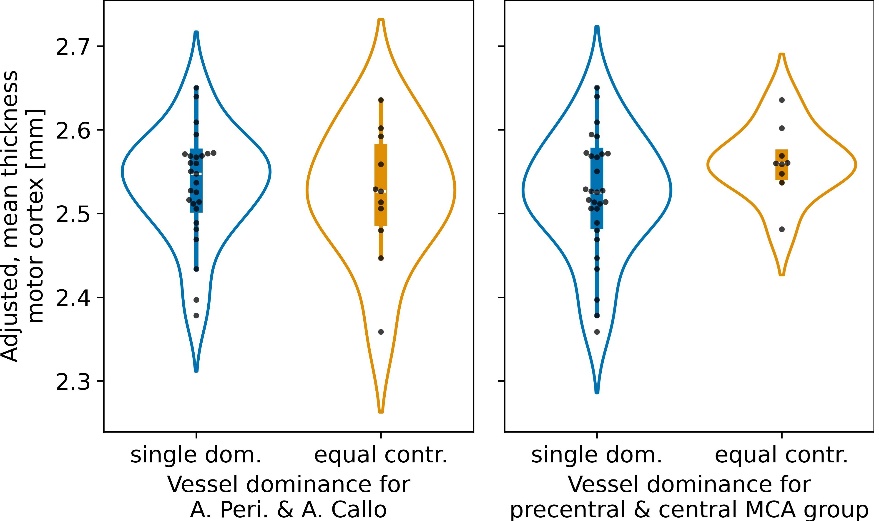


*Figure S12: Effect of vessel dominances (thresholds to group data optimized for highest agreement with Ugur et al.) on mean thickness of the motor cortex. Group comparisons are shown as box plots with swarm and violin plots overlaid. No test returned a significant group difference.*

In line with the equidistant thresholds used in the main manuscript, significant differences in motor cortex thickness due to vessel dominances were assessed with thresholds optimized for highest agreement with Ugur et al.^1^ (see Fig. S12). Models including ACA branch dominance or MCA group dominance did not significantly improve fit over the reduced model [ACA VDom: F(1, 34) = 0.234, p = 0.632; MCA VDom: F(1, 34) = 2.406, p = 0.130]. While the adjusted R² values decreased for full ACA VDom model compared to the reduced model (0.340 vs 0.354), it increased when including MCA VDom (0.379 vs 0.354). Mean cortical thickness for single vessel dominance versus equal contribution was 2.534 +/- 0.064 mm vs 2.523 +/- 0.078 mm and 2.521 +/- 0.072 mm vs. 2.561 +/- 0.042 mm for the ACA VDom and MCA VDom, respectively

Table S7: Comparison of full and reduced model for ACA branch dominance, i.e. A. pericallosal and A. callosomarginalis, using the thresholds derived from Ugur et al. Dominance was either rated as single vessel dominance or equal contribution.

| Full model: mean_thickness_motor ~ scan_age + sex + dom_peri_callo | | | | | | |
| --- | --- | --- | --- | --- | --- | --- |
|  | Coef. | Std.Err. | z | P>\|z\| | [0.025 | 0.975] |
| Intercept | 2.533 | 0.071 | 35.671 | 1.104E-278 | 2.393 | 2.672 |
| sex[T.m] | -0.117 | 0.030 | -3.898 | 9.713E-5 | -0.176 | -0.058 |
| dom_peri_callo[T.single dom.] | 0.013 | 0.031 | 0.426 | 0.670 | -0.047 | 0.073 |
| scan_age | 0.000 | 0.003 | 0.183 | 0.854 | -0.005 | 0.006 |
| Reduced model: mean_thickness_motor ~ scan_age + sex | | | | | | |
|  | Coef. | Std.Err. | z | P>\|z\| | [0.025 | 0.975] |
| Intercept | 2.531 | 0.072 | 35.282 | 1.093E-272 | 2.390 | 2.671 |
| sex[T.m] | -0.118 | 0.030 | -3.916 | 9.005E-5 | -0.176 | -0.059 |
| scan_age | 0.001 | 0.003 | 0.335 | 0.738 | -0.004 | 0.006 |
| Model Comparison via ANOVA | | | | | | |
|  | df_resid | ssr | df_diff | ss_diff | F | Pr(>F) |
| Reduced Model | 35.000 | 0.169 | 0.000 |  |  |  |
| Full Model | 34.000 | 0.168 | 1.000 | 0.001 | 0.234 | 0.632 |

Table S8: Comparison of full and reduced model for MCA group dominance, i.e. central and precentral group, using the thresholds derived from Ugur et al. Dominance was either rated as single vessel dominance or equal contribution.

| Full model: mean_thickness_motor ~ scan_age + sex + dom_central_precentral | | | | | | |
| --- | --- | --- | --- | --- | --- | --- |
|  | Coef. | Std.Err. | z | P>\|z\| | [0.025 | 0.975] |
| Intercept | 2.553 | 0.069 | 37.190 | 9.803E-303 | 2.418 | 2.687 |
| sex[T.m] | -0.123 | 0.030 | -4.139 | 3.487E-5 | -0.181 | -0.065 |
| dom_central_precentral[T.single dom.] | -0.041 | 0.021 | -1.927 | 0.054 | -0.082 | 0.001 |
| scan_age | 0.001 | 0.003 | 0.500 | 0.617 | -0.004 | 0.006 |
| Reduced model: mean_thickness_motor ~ scan_age + sex | | | | | | |
|  | Coef. | Std.Err. | z | P>\|z\| | [0.025 | 0.975] |
| Intercept | 2.531 | 0.072 | 35.282 | 1.093E-272 | 2.390 | 2.671 |
| sex[T.m] | -0.118 | 0.030 | -3.916 | 9.005E-5 | -0.176 | -0.059 |
| scan_age | 0.001 | 0.003 | 0.335 | 0.738 | -0.004 | 0.006 |
| Model Comparison via ANOVA | | | | | | |
|  | df_resid | ssr | df_diff | ss_diff | F | Pr(>F) |
| Reduced Model | 35.000 | 0.169 | 0.000 |  |  |  |
| Full Model | 34.000 | 0.158 | 1.000 | 0.011 | 2.406 | 0.130 |

## **Radius of supplying vessel**

The radii of the manually segmented vessel were estimated by fitting 2D Gaussian’s to the cross-sections of the vessel lumen along the centerline of each artery. Using these estimates, we evaluate how the vessel radius is change with distance from the motor cortex and if the vessel radii in proximity to the motor cortex differ between vessel patterns, i.e. if the number supplying vessels is associated with a difference in vessel radii.

**Vessel radius estimation**

Using a distance transform of the manual vessel segmentation would discretize radii estimate to effectively multiples of the voxel-size. For a more fine-graded approach we performed the following steps: (i) Compute the centerline of the segmented vessels. (ii) Per centerline voxel the directions along and across the vessel are found, i.e. the tangent vector and its orthogonal counterparts. All centerline voxels with in a 5x5x5 patch centered around the current voxel where included in a single-value decomposition (SVD) to find the first eigenvector, i.e. tangent vector, as well as the second and third eigenvectors, i.e. vectors orthogonal to the vessel’s trajectory. (iii) Using the orthogonal vectors, the cross-section of the vessel lumen at the current centerline voxel was computed. Thus, a 5x5 patch of the original MPRAGE intensity values linearly interpolated at along the orthogonal directions was generated capture in the 2D lumen intensity profile. (iv) Fit a 2D Gaussian to the 5x5 intensity profile to find the standard deviations along the two orthogonal directions. (v) Convert the standard deviation to full-width-half-maximum (FWHM) estimates and approximate the radius per centerline voxel as the geometric mean of the two FWHM estimates.

**Vessel radius as a function of distance to motor cortex**

To determine how vessel radius changes as arteries approach the motor cortex, we plotted the average radius per hemisphere against the distance to the motor cortex. Distances were binned into [0, 3) mm, [3, 6) mm, [6, 10), and [10, ∞) respectively, and beyond the average radius per hemisphere, the number of centerline voxel within this distance bin plotted (see Fig. S13).


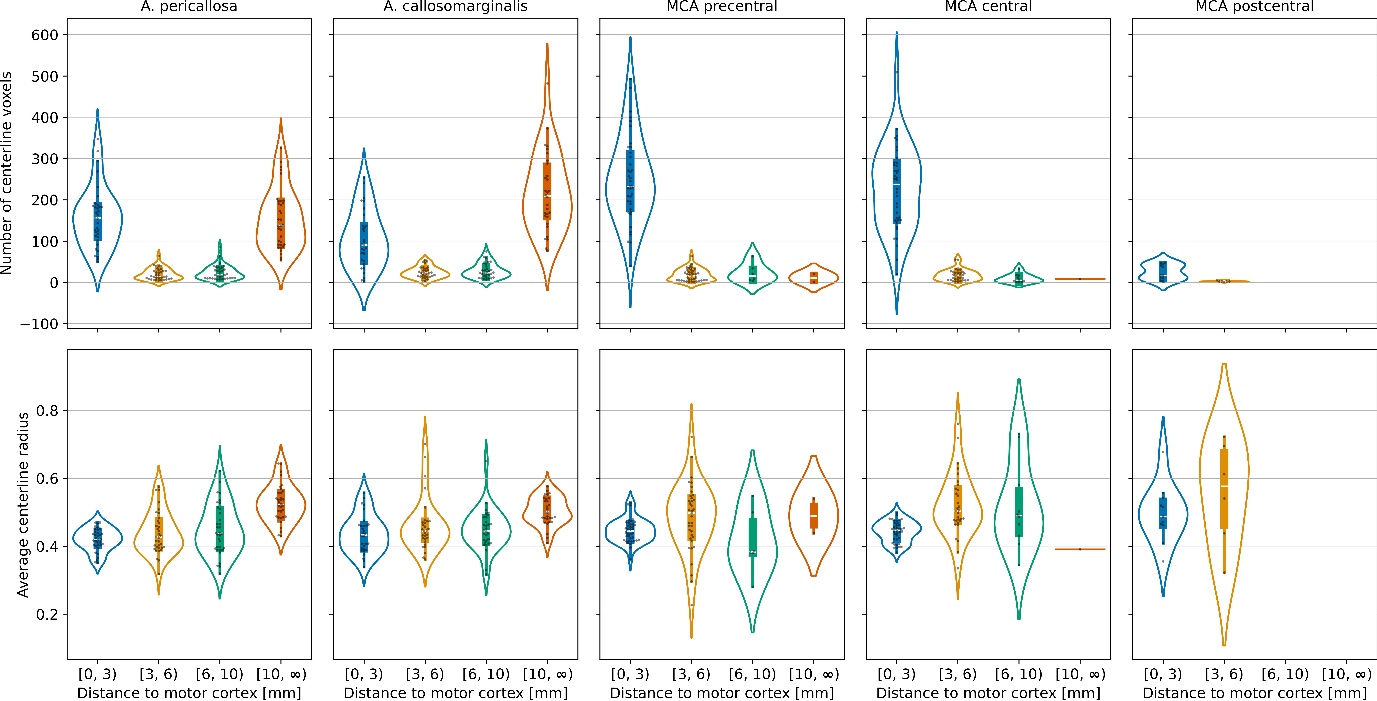


Figure S13: The number of centerline voxel and average radius investigated as a function to distance to the motor cortex for each artery of interest

Fig. S13 shows that the majority of segmented vessels of the MCA groups is in close proximity to the motor cortex, i.e. within 3 mm distance. For the ACA branches a roughly half of the centerline voxels is within 3 mm from the motor cortex and the other half more than 10 mm away. The reason for the differences between MCA groups and ACA branches is that the MCA groups branch of the MCA much closer than the A. pericallosa and A. callosomarginalis branch of the ACA. Therefore, the segmentations of the ACA branches begin much further away from the motor cortex.

There is a general trend of reducing vessel radii with decreasing distance to the motor cortex. In particular for the ACA branches, is can be observed as the radius is decreasing from radius 0.525±0.055 mm at [10, ∞) mm to radius 0.419±0.033 mm at [0, 3) mm and from radius 0.502±0.041 mm at [10, ∞) mm to radius 0.436±0.056 mm at [0, 3) mm for the A. pericallosa and A. callosomarginalis, respectively.

**Vessel radius and vessel patterns**

One might suspect that if the number of arteries supplying the motor cortex is lower, thick arteries are required to provide adequate blood supply. Therefore, we grouped the average vessel radii for vessel patterns, i.e. number of supplying vessels, and tested if there are significant differences. The average vessel radius per hemisphere within 3 mm distance to the motor cortex was used to be sensitive towards the vasculature in proximity of the motor cortex and not the entire vessel which might supply further brain regions beyond the scope of this study. Further, the postcentral group is not included as it is only present in vessel patter with 5 supplying arteries.


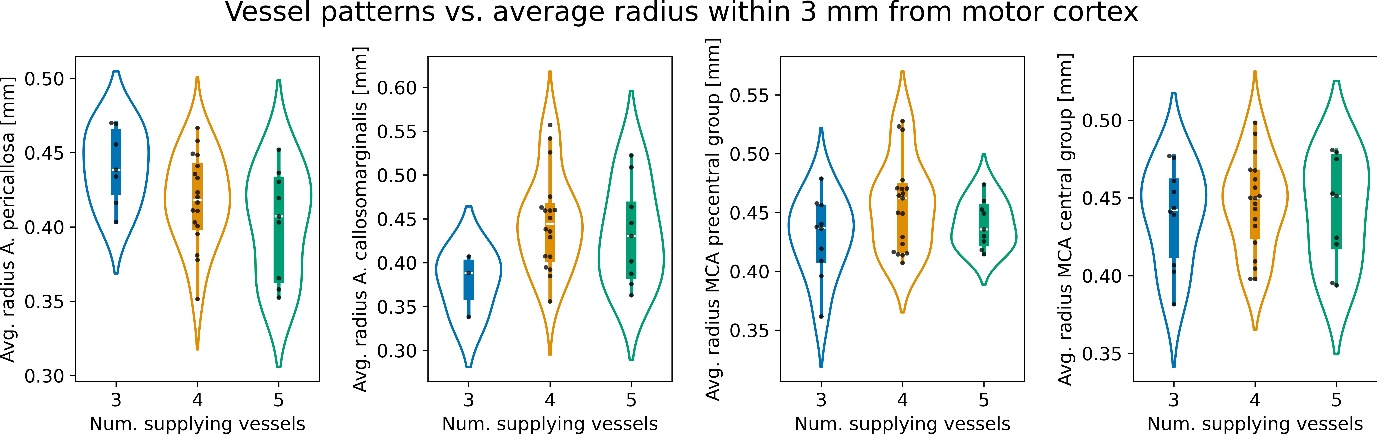


*Figure S14: Difference in average vessel radius for different vessel patterns, i.e. different number of supplying vessels.*

After Bonferroni correction for multiple comparisons, testing showed no significant group differences: F(2, 14.728) = 3.204, p = .070, ηp² = .161; χ²(2) = 3.862, p = .145; F(2, 35) = 2.266, p = .119, ηp² = .115; and F(2, 35) = 0.181, p = .835, ηp² = .010; for the radii of the A. pericallosa, A. callosomarginalis, MCA precentral group, and MCA central group, respectively.

Nevertheless, there is a trend for a small decrease in radii when comparing vessel patterns with 4 vs. 5 supplying vessels. The group average radii decreased from 4- to 5-supplying vessel pattern from 0.418±0.030 mm to 0.403±0.036 mm, from 0.447±0.054 mm to 0.433±0.057 mm, 0.456±0.038 mm to 0.440±0.020 mm, and from 0.445±0.030 mm to 0.441±0.034 mm for the A. pericallosa, the A. callosomarginalis, the MCA precentral group, and the MCA central group, respectively. For the A. pericallosa this trend extends to the 3-vessel pattern (group average radius 0.441±0.026 mm), showing a monotonic decrease in radii with increasing number of supplying vessels. For the A. callosomarginalis, hemispheres with 3 supplying vessel have considerably lower average radii (0.378±0.036 mm), but only three hemispheres are included in this group, warrant caution when interpreting this outcome.

Overall, the difference in radii was less than 0.1 mm. This shows the need for continuous radius estimates via e.g. the used Gaussian fit of the lumen cross-section instead of a distance transform of the segmentation which provides radii estimates discretized to the voxel grid. Further, it shows that the overall changes are small, and considering the number of hemispheres included in this study requires follow-up studies to verify the generalizability of the observed trend and, potentially, showing statistical significance.

## **Interhemispheric difference: vessel patterns vs. cortical thickness**

While our primary analysis revealed no significant overall differences in motor cortex thickness between the left and right hemispheres, we additionally investigated whether interhemispheric differences in motor cortex thickness were associated with differences in the number of supplying vessels, i.e. vessel pattern difference. For each participant, we computed the absolute interhemispheric difference for both motor cortex thickness and the number of supplying vessels. The absolute difference in supplying vessels was categorized into three groups (0, 1, and 2, reflecting increasing levels of asymmetry in vessel patterns) for subsequent analysis. Descriptively, the group with no difference in vessel patterns (Group 0, N=6) showed an absolute cortical thickness difference of 0.060±0.021 mm. For Group 1 (N=11), the difference was 0.053±0.033 mm, and for Group 2 (N=2), it was 0.075±0.020 mm. Although these values suggest a descriptive "U-shaped" pattern (see Fig. S15), a Kruskal-Wallis H-test revealed no statistically significant differences in absolute cortical thickness across these vessel pattern groups (H(2)=0.773, p=0.679).


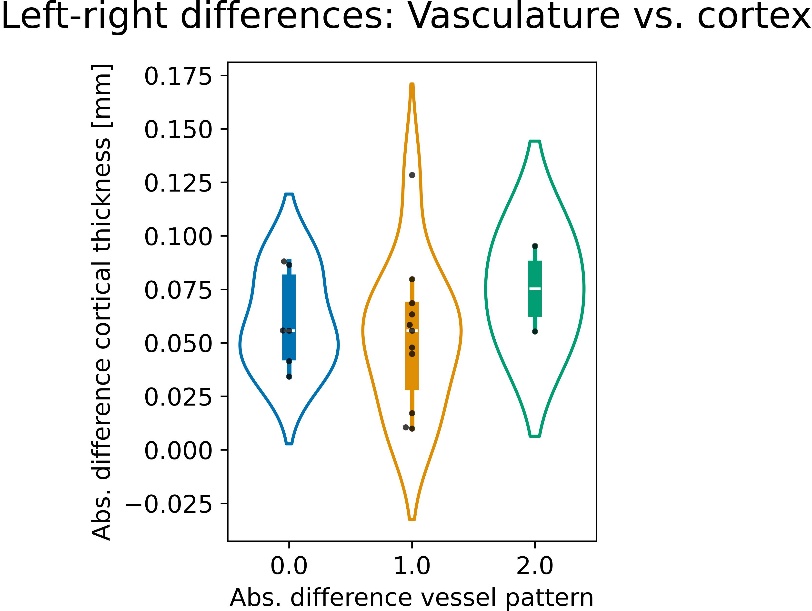


*Figure S15: Comparison of interhemispheric difference of vessel patterns, i.e. number of supplying vessels, and cortical thickness of the motor cortex.*

**Reference list**

1. Ugur, H. C. *et al.* Arterial vascularization of primary motor cortex (precentral gyrus). *Surg. Neurol.* **64 Suppl 2**, S48-52 (2005).
